# Tumour cell CD99 regulates transendothelial migration via CDC42 and actin remodelling

**DOI:** 10.1242/jcs.240135

**Published:** 2021-08-10

**Authors:** Aarren J. Mannion, Adam F. Odell, Alison Taylor, Pamela F. Jones, Graham P. Cook

**Affiliations:** Leeds Institute for Medical Research, University of Leeds School of Medicine, St. James's University Hospital, Leeds LS8 2BH, UK

**Keywords:** CD99, CDC42, Actin cytoskeleton, Transendothelial migration, Metastasis, Breast cancer

## Abstract

Metastasis requires tumour cells to cross endothelial cell (EC) barriers using pathways similar to those used by leucocytes during inflammation. Cell surface CD99 is expressed by healthy leucocytes and ECs, and participates in inflammatory transendothelial migration (TEM). Tumour cells also express CD99, and we have analysed its role in tumour progression and cancer cell TEM. Tumour cell CD99 was required for adhesion to ECs but inhibited invasion of the endothelial barrier and migratory activity. Furthermore, CD99 depletion in tumour cells caused redistribution of the actin cytoskeleton and increased activity of the Rho GTPase CDC42, known for its role in actin remodelling and cell migration. In a xenograft model of breast cancer, tumour cell CD99 expression inhibited metastatic progression, and patient samples showed reduced expression of the *CD99* gene in brain metastases compared to matched primary breast tumours. We conclude that CD99 negatively regulates CDC42 and cell migration. However, CD99 has both pro- and anti-tumour activity, and our data suggest that this results in part from its functional linkage to CDC42 and the diverse signalling pathways downstream of this Rho GTPase.

This article has an associated First Person interview with the first author of the paper.

## Abstract (precis)

**Summary:** Tumour cell surface CD99 regulates metastatic progression *in vivo* and inhibits the transendothelial migration of cancer cells by regulating the Rho GTPase CDC42 and actin remodelling.

## INTRODUCTION

Ninety percent of cancer deaths are attributable to metastatic progression (Lambert et al., 2017). The interaction between disseminating metastatic cancer cells and cells of the endothelium is an important step in the metastatic cascade (Lambert et al., 2017). From the primary tumour, migrating cancer cells intravasate, gaining access to the lymphatic and haematogenous vasculature (Strilic and Offermanns, 2017). Once in the circulation, those cancer cells that survive shear flow, anoikis and immune surveillance may reach distant sites where they can extravasate and potentially seed a metastasis (Lambert et al., 2017). The intravasation and extravasation of tumour cells require intimate interactions between cancer cells and endothelial cells (ECs), with both cell types contributing to the initial adhesive interactions and subsequent transendothelial migration (TEM) (Reymond et al., 2013). Leucocytes undergo TEM when extravasating from blood to infected tissues as part of the inflammatory process, and this extensively studied mechanism provides a model for how cancer cell TEM might operate during metastasis (Madsen and Sahai, 2010; Vestweber, 2015).

The type 1 transmembrane receptor CD99 is expressed in a variety of tissues, including haematopoietic lineage cells and EC (Pasello et al., 2018). Amongst its reported functions, CD99 regulates the adhesion and TEM of haematopoietic cells during inflammation (Lou et al., 2007; Schenkel et al., 2002). In the endothelium, CD99 resides at the EC borders in a complex with ezrin, soluble adenylyl cyclase (sAC) and cAMP-dependent protein kinase, also known as protein kinase A or PKA (Watson et al., 2015). EC CD99 is also localised to the lateral border recycling compartment (LBRC), an intracellular compartment contiguous with the plasma membrane and located proximal to EC-cell junctions (Mamdouh et al., 2003; Watson et al., 2015). During TEM, CD99 on the leucocyte and endothelium undergo homophilic interactions that facilitate the movement of the leucocyte between the EC junctions (Schenkel et al., 2002; Watson et al., 2015). Engagement of endothelial CD99 triggers sAC, generating cAMP, which activates PKA and enables the rapid turnover of CD99 from the LBRC to the EC junction to facilitate transmigration (Watson et al., 2015). Although well established in leucocyte TEM, the involvement of CD99 in cancer progression remains enigmatic (Manara et al., 2018). For example, CD99 is highly expressed in several haematopoietic malignancies and in Ewing's sarcoma. For the latter, high CD99 expression is associated with increased migration, increased tumour growth and greater metastasis (Kreppel et al., 2006; Rocchi et al., 2010). However, in other cancers (such as osteosarcoma and gastric cancer), decreased CD99 expression is associated with tumour progression (Manara et al., 2018). Thus, despite an established role for CD99 in leucocyte TEM, the function of CD99 in tumour cell TEM and the metastatic pathway is currently ill-defined.

Members of the Rho GTPase family, in particular RhoA, Rac1 and CDC42, control many cellular responses, including cytoskeletal organisation and migratory activity (Haga and Ridley, 2016; Hall, 2012). The Rho GTPases cycle between an inactive guanosine diphosphate (GDP)-bound conformation and an active guanosine triphosphate (GTP)-bound conformation, the latter interacting with downstream effector molecules. This on/off switch is regulated by guanine nucleotide exchange factors (GEFs) that enhance the replacement of GDP with GTP in response to extracellular signals arising from cell surface receptors (Schiller, 2006; Yu and Brown, 2015). Furthermore, activity is negatively regulated by GTPase activating proteins (GAPs), which enhance the intrinsic GTPase activity, and by guanine nucleotide dissociation inhibitors (GDIs), which sequester the GTPase away from membrane proximal signalling events (Haga and Ridley, 2016). In its active GTP-bound confirmation, CDC42 acts together with N-WASP to activate the ARP2/3 complex, facilitating the nucleation of actin and regulating cytoskeletal organisation (Ma et al., 1998; Rohatgi et al., 1999). The TEM of leucocytes requires cytoskeletal rearrangements in both the migrating cell and the endothelium, and Rho GTPases have been implicated in this process (Heasman and Ridley, 2008; Kouklis et al., 2004; Vicente-Manzanares and Sánchez-Madrid, 2004). In addition, Rho GTPases regulate many pathways important in the malignant phenotype (Haga and Ridley, 2016), and CDC42 activity (but not RhoA or Rac1) is required for TEM and metastatic progression in prostate and breast cancer models (Reymond et al., 2012).

Breast cancer metastasises to multiple sites, most frequently to the bones, lung, brain and liver, and patients with triple negative breast cancer, lacking expression of the oestrogen receptor (ER), the progesterone receptor (PR) and HER2, have fewer treatment options and a greater risk of metastasis (Garrido-Castro et al., 2019; Riggio et al., 2021). Similarly, bone metastases of prostate cancer are typically treated with palliative care only (Berish et al., 2018). Both breast and prostate cancer metastasis models are intensively studied, with TEM identified as a critical and potentially targetable pathway (Berish et al., 2018; Bos et al., 2009; Kang et al., 2003; Minn et al., 2005a,b; Neve et al., 2006; Reymond et al., 2012). Here, we show that CD99 regulates metastatic progression *in vivo* and that CD99 negatively regulates CDC42 activity, linking CD99 to cytoskeletal reorganisation, migration and TEM, key components of the metastatic pathway.

## RESULTS

### Tumour cell CD99 regulates adhesion and transendothelial migration

**Fig. 1.**
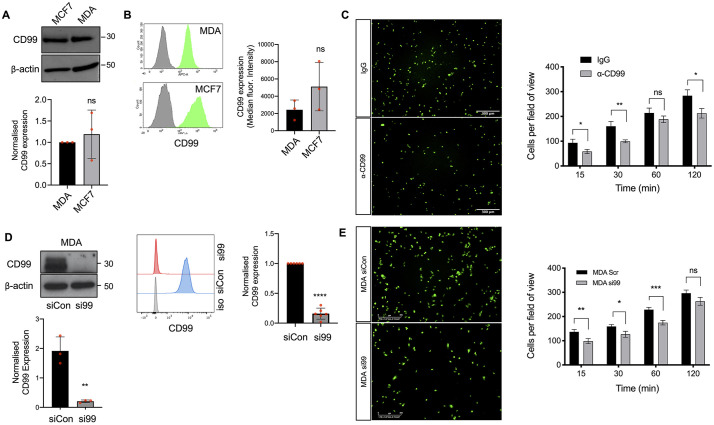
**CD99 regulates breast cancer adhesion to endothelial cells.** (A) Expression of total CD99 protein expression determined by western blotting in breast cancer cell lines MCF7 and MDA-MB-231 (MDA) using anti-CD99 antibody and anti-β actin as a loading control. The graph shows quantification of CD99 protein expression in MCF7 and MDA-MB-231 normalised to the β actin loading control. *n*=3 independent experiments. (B) Expression of cell surface CD99 on MCF7 and MDA-MB-231 determined by flow cytometry (in green) compared to the isotype control (grey); the graph shows quantification normalised to isotype controls. *n*=3 independent experiments. (C) Representative images and quantification of the adhesion of Cell Tracker Green (CTG)-labelled MDA-MB-231 cells adhering to confluent HUVEC monolayers pre-treated with anti-CD99 blocking antibody (1 μg/ml) or an IgG control (shown at the 30-min time point). Unbound cells were washed away with PBS before fixation in 4% PFA and quantification of bound cells. *n*=3 independent experiments. (D) MDA-MB-231 cells were transiently transfected with siRNA targeting CD99 (si99) or a control siRNA (siCon), and CD99 expression was determined using western blotting and flow cytometry 72-96 h post-transfection. For the blot, the graph shows CD99 expression relative to the β actin loading control in the presence of the siRNA molecules. For the flow cytometry, quantification was performed by comparing CD99 expression in the siCon-treated cells compared to si99-treated cells. *n*=3 independent experiments and *n*=6 independent experiments for western blots and flow cytometry, respectively. (E) MDA-MB-231 cells were transfected as in D for 72 h before CTG labelling and left to adhere to confluent HUVEC monolayers for indicated time points. Unbound cells were washed away with PBS before fixation, imaging and quantification of bound MDA cells using ImageJ (the images show the 60-min time points). *n*=3 independent experiments. Data are mean±s.d. (A,B) or s.e.m. (C-E). *P<0.05; **P<0.005; ***P<0.0005; ns, not significant [unpaired two-tailed Student's *t*-test (with multiple comparisons in C)].

Leucocytes use CD99 to cross endothelial barriers (Lou et al., 2007; Schenkel et al., 2002; Watson et al., 2015), suggesting that tumour cell CD99 might regulate TEM during metastasis. We determined the expression of CD99 by the cell lines MCF7 and MDA-MB-231, which differ in their invasive potential; MCF7 is a non-invasive ER^+^ breast cancer cell line, whereas MDA-MB-231 is a highly invasive triple negative breast cancer cell line (Neve et al., 2006). Immunoblotting and flow cytometry demonstrated that expression of CD99 was not significantly different between two cell lines (Fig. 1A,B), and we subsequently focused on the role of CD99 in the invasive activity of MDA-MB-231 cells, a cell line frequently used to dissect pathways of metastasis, including the analysis of TEM (Bos et al., 2009; Kang et al., 2003; Minn et al., 2005a,b; Neve et al., 2006; Reymond et al., 2012). Adhesion of fluorescently labelled MDA-MB-231 to an EC monolayer was inhibited by an anti-CD99 antibody (Fig. 1C), consistent with earlier studies identifying CD99 as an adhesion molecule (Gelin et al., 1989). ECs also express CD99 (Lou et al., 2007) and we used RNAi to determine whether it was the tumour or EC CD99 that regulated these adhesion events. Expression of CD99 was reduced in both tumour and EC using siRNA (Fig. 1D; Fig. S1A). Neither the survival nor *in vitro* proliferation of MDA-MB-231 cells were significantly impacted by CD99 depletion (Fig. S1B,C). However, CD99-depleted MDA-MB-231 cells showed significantly reduced adhesion to EC monolayers at early time points (Fig. 1E). In contrast, loss of EC CD99 did not significantly decrease tumour cell adhesion and, at a late time point, adhesion was significantly increased, revealing a non-equivalent role for CD99 in these tumour cells and interacting endothelium (Fig. S1D).

**Fig. 2.**
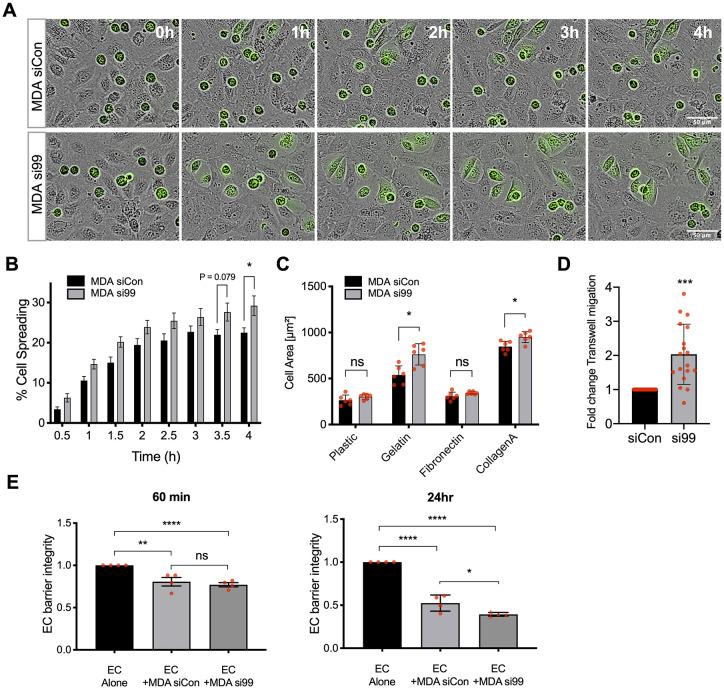
**Breast cancer migration and invasion is regulated by CD99.** (A) MDA-MB-231 TEM and intercalation determined by live cell imaging. Control (siCon) or CD99 (si99) siRNA-treated MDA-MB-231 cells were CTG labelled and seeded onto HUVEC monolayers, and intercalation/spreading was captured using live cell imaging. Images were taken every 5 min for 4 h using a 20× objective. (B) Quantification of data in panel A, indicating the percentage of MDA-MB-231 cells that have undergone spreading/intercalation as a percentage of total cells*. n*=3 independent experiments. (C) Cell area of CD99 siRNA-transfected MDA-MB-231 cells bound to indicated ECM components for 30 min was determined using Columbus image analysis software. Data were collected from multiple cells from two images from *n*=3 independent experiments. (D) Quantification of TEM of CD99 siRNA-treated MDA-MB-231 cells determined using the modified Boyden transwell insert (3 µm pore size). HUVEC cells were seeded to the upper chamber of transwell inserts for 24 h before CTG-labelled siRNA-treated MDA-MB-231 cells were seeded on top of EC monolayers for 18 h before fixation and imaging following transmigration. We used two transwells per condition and analysed three images per transwell per independent experiment using ImageJ. *n*=3 independent experiments. (E) MDA-MB-231 cells treated with control or CD99 siRNA were seeded onto confluent HUVEC monolayers and changes in impedance were recorded for 24 h at 37°C under 5% CO_2_. Data are presented as fold change in barrier integrity compared to HUVEC monolayers alone at indicated time points. *n*=4 independent experiments. Data are mean±s.e.m. (B,D,E) or s.d. (C). Statistical significance was determined using an unpaired *t*-test with multiple comparisons (B-D) or one-way ANOVA with Tukey's multiple comparison (E) (*P<0.05; **P<0.005; ***P<0.0005; ****P<0.0001; ns, not significant).

Following adhesion, extravasating cells undergo TEM by crawling between, or sometimes through, ECs (Reymond et al., 2013). Time-lapse microscopy was used to monitor the interactions of fluorescently labelled MDA-MB-231 cells with an unlabelled EC monolayer (Fig. 2). Tumour cells interacting with the endothelium altered their morphology from spherical structures (with high circularity) towards a spread and more flattened appearance, and depletion of tumour cell CD99 increased this spreading phenotype, an effect that was statistically significant at 4 h (Fig. 2A,B). The human prostate cancer cell line PC3 is also used to analyse cancer cell TEM and metastatic pathways (Berish et al., 2018; Reymond et al., 2012); this cell line expresses CD99 and, upon CD99 depletion, also demonstrates increased spreading on an EC monolayer (Fig. S2). In the breast cancer model, ∼30% of MDA-MB-231 cells depleted of CD99 were flattened onto the endothelial monolayer after 4 h, compared to ∼20% of the cells treated with control siRNA (Fig. 2B). The increased spreading of CD99 depleted cells was independent of interaction with ECs, with greater spreading also occurring on gelatin and collagen A matrices (Fig. 2C). Importantly, transwell assays showed that CD99 depletion resulted in a twofold enhancement of TEM activity compared to the control siRNA-treated cells (Fig. 2D), demonstrating that the increased spreading of the tumour cells on the EC monolayer was associated with increased TEM activity. The invasion of the endothelial barrier requires EC-EC junctions to be broken and the resultant loss in monolayer integrity can be assessed by measuring the reduction in electrical resistance across the monolayer (Rahim and Üren, 2011). Addition of tumour cells to an EC monolayer resulted in a significant loss of endothelial barrier integrity at both 1 h and 24 h, consistent with TEM activity (Fig. 2E). Tumour-mediated loss of barrier integrity was independent of CD99 expression at the 1 h time point. However, CD99-depleted MDA-MB-231 cells displayed a significantly enhanced ability to disrupt the endothelial barrier at the 24 h time point (Fig. 2E).

**Fig. 3.**
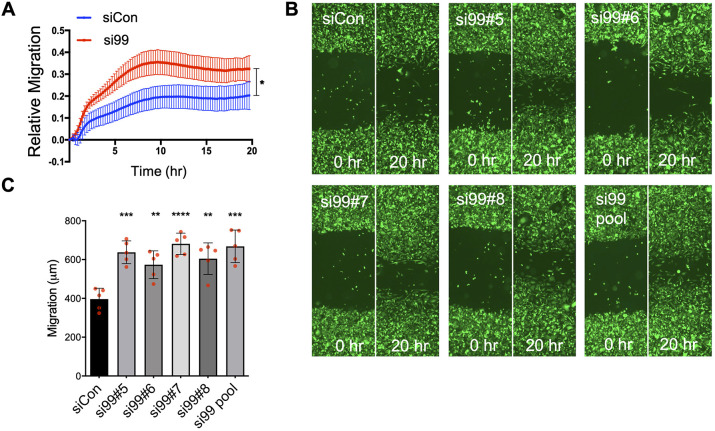
**CD99 suppresses breast cancer migration *in vitro*.** (A) Real-time migration of CD99 siRNA-treated MDA-MB-231 cells determined using the modified Boyden chamber CIM plates and xCelligence RTCA. Changes in impedance are indicative of the number of cells that have migrated. Impedance was measured every 15 min for 24 h. *n*=4 independent experiments. (B) Representative images of scratch wound migration assay. MDA-MB-231-GFP cells were treated with the indicated individual CD99 siRNA duplexes, pooled CD99 siRNA or control siRNA for 72 h and seeded at equal density into 96-well plates until confluent. Cells were serum starved for 2-3 h before ‘wounding’ using a Wound Maker tool (Essen Bioscience). Migration of siRNA-treated MDA-MB-231 cells was imaged at 0 and 20 h and quantified using ImageJ. (C) Quantification of B, from *n*=5 independent replicates. Data are mean±s.e.m. *P<0.05, **P<0.005, ***P<0.0005, ****P<0.0001 [analysis of covariance (A) and one-way ANOVA with Tukey's multiple comparison (C)].

The ability of inflammatory cells or tumour cells to intercalate and cross the endothelium is a migratory function. Real time migration assays (using real-time cell analyser modified Boyden chambers) showed that CD99 depletion significantly increased the migratory activity of MDA-MB-231 cells (Fig. 3A), consistent with the inhibitory effects of tumour cell CD99 expression on intercalation, cell spreading and endothelial barrier function (Fig. 2A-E). To test this phenotype in more detail, we performed end-point migration assays (using scratch wound repair) with the siRNA pool and its individual siRNA molecules, employing a GFP-expressing variant of MDA-MB-231. All four individual siRNA molecules from the pool reduced CD99 expression, as determined by fluorescence microscopy and imaging cytometry (Fig. S3). Employing these cells in the scratch wound assay revealed that all siRNAs targeting CD99 resulted in significantly increased migration compared to the control siRNA (Fig. 3B,C).

### CD99 regulates cytoskeletal dynamics and CDC42 activity

**Fig. 4.**
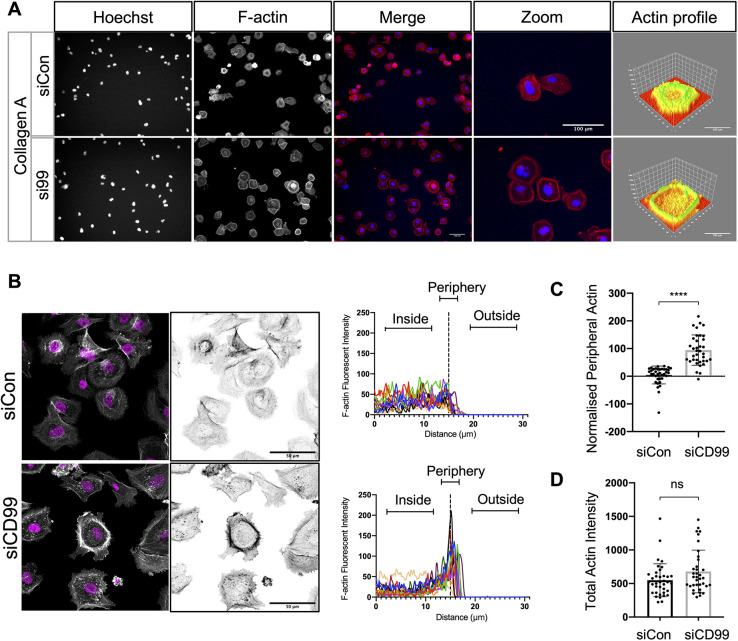
**Breast cancer actin dynamics are modulated by CD99 depletion.** (A) Control or CD99 siRNA-treated MDA-MB-231 cells (48 h post transfection) were re-plated to collagen type 1 coated plates and allowed to adhere for 30 min. Cells were then stained with Texas Red phalloidin (for F-actin) before imaging using an Operetta HTS imager. Three-dimensional actin distribution plots were generated using ImageJ ‘3D surface plot’ analysis. Images are representative of *n*=3 independent experiments. (B) As in panel A, siRNA-treated cells were imaged using a LSM 700 confocal microscope, and actin intensity was analysed using ImageJ. Trace plots indicate actin intensity inside the cell, at the cell periphery and outside the cell (as indicated) overlayed for 12 individual cells (four cells each from three random fields of view) from one experiment. Data from two additional experiments are shown in Fig. S4. (C) Quantification of B, where values of actin intensity at the cell periphery were normalised to the cytoplasmic actin of 36 cells from *n*=3 independent experiments. (D) Quantification of total actin intensity of control or CD99 siRNA-treated MDA-MB-231 cells shown in B and C. Values of total actin intensity of 36 cells from *n*=3 independent experiments. ****P<0.00005; ns, not significant (unpaired two-tailed *t*-test).

Leucocyte TEM requires substantial cytoskeletal remodelling (Vicente-Manzanares and Sánchez-Madrid, 2004), and similar mechanisms operate in extravasating cancer cells (Madsen and Sahai, 2010). We plated RNAi-treated MDA-MB-231 cells onto collagen A (upon which the enhanced spreading phenotype of CD99-depleted cells mirrored that found on the ECs; Fig. 2A-C) and analysed their actin organisation using immunofluorescence microscopy; the flattened morphology of the CD99 depleted cells was accompanied by actin rearrangement, with its accumulation at the cell periphery (Fig. 4A). We quantified actin distribution using a method adapted from a study of cell-cell interactions (Nakajima et al., 2017); actin content was analysed via pixel intensity along an axis starting inside the cell, crossing the cell periphery and extending into the extracellular space (Fig. 4B). This demonstrated the significant accumulation of peripheral actin upon CD99 depletion compared to control siRNA-treated cells (Fig. 4B,C; Fig. S4A). However, the total actin content was not significantly different between control and CD99-depleted cells (Fig. 4D). We did not observe the enhanced cell spreading phenotype when si99-treated cells were plated on fibronectin (Fig. 2C), and actin distribution on this substrate was unchanged by CD99 depletion (Fig. S4B).

**Fig. 5.**
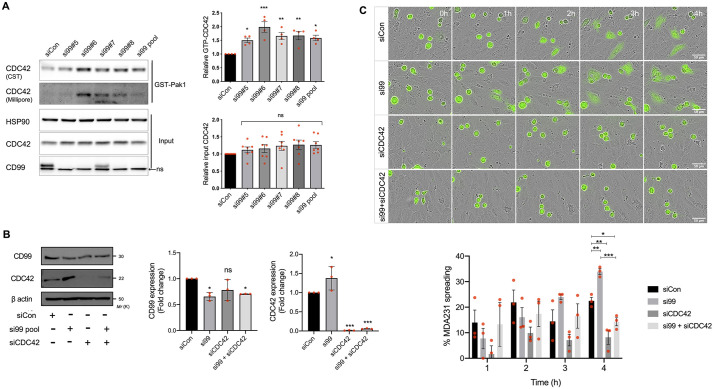
**Breast cancer TEM is negatively regulated by CD99 through suppression of CDC42 activity.** (A) MDA-MB-231 cells were transfected with control, pooled or indicated individual CD99 siRNA molecules for 72 h, and lysates were subject to immunoprecipitation with PAK1-PDB beads and subsequent electrophoresis and blotting to determine GTP-bound CDC42. Precipitated GTP-bound CDC42 was analysed using two different antibodies as indicated. Input control lysates, which had not been subjected to immunoprecipitation with PAK1-PDB beads were electrophoresed alongside pulldown samples to determine total CDC42 protein expression. A non-specific band was present in the CD99 immunoblots (nsb). The graphs (right) show quantification (determined using ImageJ) of active GTP-bound CDC42 and total CDC42; the former was normalised to total CDC42 and the latter to HSP90. *n*=4 independent experiments for active CDC42 and *n*=7 for total CDC42. (B) siRNA-mediated knockdown of CD99, CDC42 and co-depletion of CD99 and CDC42 72 h post transfection was determined by western blotting using anti-CD99, anti-CDC42 and anti-β actin antibodies as shown. Quantification (right) shows expression of CD99 and CDC42 normalised to β-actin loading control relative to control siRNA-treated cells. *n*=3 independent experiments. (C) MDA-MB-231 TEM and intercalation determined by live cell imaging. Control, CD99, CDC42 or CD99 and CDC42 siRNA-treated MDA-MB-231 cells were CTG-labelled and seeded onto HUVEC monolayers, and intercalation/spreading was captured using live cell imaging. Images were taken every 5 min for 4 h using a 20× objective. The graph (right) shows the quantification of data in C, indicating the percentage of MDA-MB-231 cells that have undergone spreading/intercalation as a percentage of total cells*. n*=3 independent experiments. Data are mean±s.d. (A,B) or s.e.m. (C). Statistical significance was determined using one-way ANOVA and Dunnett's multiple comparison test (A), one-way ANOVA (B) and one-way ANOVA with Tukey's multiple comparison test (C). *P<0.05; **P<0.005; ***P<0.0005; ns, not significant.

Changes in actin distribution are mediated by members of the Rho GTPase family (Haga and Ridley, 2016; Hall, 2012). Furthermore, TEM and metastasis of both MDA-MB-231 and PC3 cell lines is dependent upon CDC42 (Reymond et al., 2012), suggesting that CD99 might be linked to actin rearrangement and the altered TEM phenotype via this Rho GTPase. Active CDC42 (with bound GTP) interacts with and activates the serine/threonine kinase PAK1, and hence bead-bound PAK1 can be used to selectively pull down active CDC42, which can then be identified using anti-CDC42 antibodies (Benard et al., 1999). Using this approach, we found that CD99 depletion in MDA-MB-231 cells resulted in an increase in active CDC42 when either the siRNA pool or the individual members of the pool were used, and quantification demonstrated a statistically significant increase in GTP-CDC42 with all of the siRNA molecules (Fig. 5A). This increase in CDC42 activity is consistent with the increased migration of cells following CD99 depletion with this panel of siRNA molecules (Fig. 3B,C). Interestingly, total CDC42 levels were also increased following CD99 depletion with these siRNAs, but this was not statistically significant across the group (Fig. 5A).

To verify a functional interaction between CD99 and CDC42 we performed RNAi against tumour cell CD99 and CDC42, individually and in combination. The expression of CD99 was unaffected by CDC42 knockdown, whereas total CDC42 expression again demonstrated a small increase when CD99 was depleted using the siRNA pool; quantification and paired statistical testing demonstrated a small but significant increase in total CDC42 in si99-treated cells compared to the control siRNA treatment (Fig. 5B). Importantly, the spreading of tumour cells on ECs was inhibited by the depletion of CDC42, and the enhanced spreading of tumour cells seen upon CD99 depletion was also CDC42 dependent, with statistically significant differences in phenotype observed at the 4-h time point (Fig. 5C). These results are consistent with a pathway in which tumour cell surface CD99 regulates cytoskeletal dynamics in a CDC42-dependent manner; specifically, CD99 expression negatively regulates CDC42 activity.

### Tumour cell CD99 negatively regulates metastasis *in vivo*

**Fig. 6.**
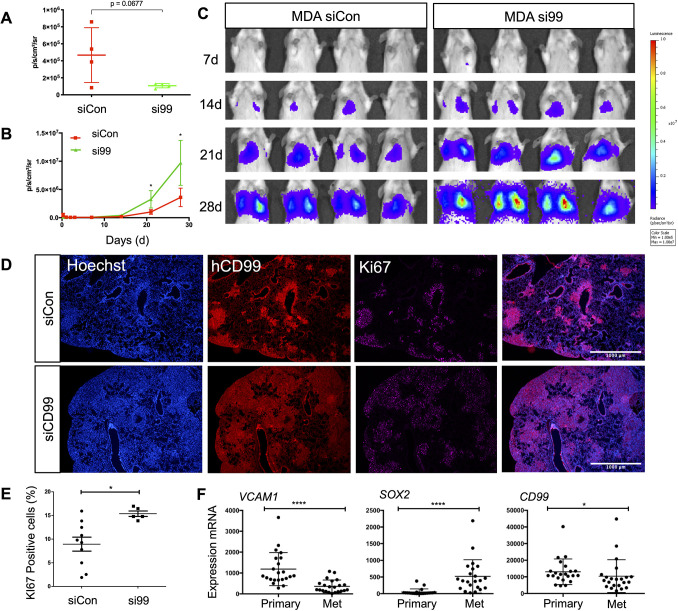
**CD99 suppresses the metastatic phenotype of breast cancer *in vivo.*** Luciferase expressing MDA-MB-231 cells were transfected with control or CD99 siRNA and 72 h later, 5×10^5^ cells/mouse were injected into the main tail vein of female CB17-SCID mice (aged 6-8 weeks). (A) Quantification of bioluminescent imaging from 6 h post tail vein injection. *n*=4 mice per group. (B) Quantification of bioluminescent imaging from each time point over the course of 28 days. *n*=4 mice per group. (C) For imaging, D-luciferin substrate was injected, and anaesthetised mice from each group were scanned using an IVIS at the indicated times. Images from additional time points are shown in Fig. S5. (D) Representative images of lung tissue from mice injected with control or CD99 siRNA-treated MDA-MB-231 cells. Lungs were isolated 4 weeks post injection and analysed by staining with Hoechst (to detect all cells), anti-human CD99 antibody (to detect the human tumour cells) and anti-Ki67 antibody (to detect proliferating cells). Merged images are also shown. Scale bars: 1000 µm. (E) Quantification (using ImageJ) of Ki67 staining as a percentage of total Hoechst nuclei. (F) Gene expression in primary and matched brain tumour metastases from 22 breast cancer patients (Varešlija et al., 2019). Data for *VCAM1*, *SOX2* and *CD99* gene expression in the primary and metastatic (Met) samples were analysed using a Wilcoxon matched pairs signed rank test (*P<0.05, ****P<0.00005). Data are mean±s.d. In A and B, statistical significance was determined using an unpaired two-tailed Student's *t*-test.

These results highlight a complex role for CD99 in tumour cell TEM; CD99 is coupled to cytoskeletal organisation and positively regulates tumour-EC adhesion, but negatively regulates post-adhesion events, including cell migration. Such complexity reflects the multistage nature of tumour cell TEM and is consistent with previous findings suggesting either pro- or anti-tumour activity for CD99 (Manara et al., 2018). To determine the overall contribution of CD99 expression to metastasis and tumour progression we used an *in vivo* model. When injected into the tail vein of immunodeficient mice, the MDA-MB-231 cell line preferentially metastasises to the lungs. We used luciferase-expressing MDA-MB-231 cells, transfected with either control or CD99 siRNA molecules, and assayed tumour burden repeatedly over 4 weeks using *in vivo* luciferase imaging. Both control-treated and CD99-depleted tumour cells were detectable in the lungs of most mice 6 h post-injection, and all animals developed lung tumours over the 4 weeks of the assay (Fig. S5). However, luciferase signals in the lungs of animals injected with control tumour cells exceeded signals from CD99-depleted cells by ∼fivefold at the 6-h time point (*P*=0.067; Fig. 6A). Despite seeding less efficiently than the control-treated cells, the CD99-depleted cells showed greater tumour growth in the longer term, with statistically significant differences in luciferase signals observed at the 3- and 4-week time points (Fig. 6B,C). Depletion of CD99 in the implanted cells was via transient transfection of siRNA, allowing CD99 expression to be used at the 4 week time point to judge tumour burden in these animals; mice implanted with CD99-depleted tumours showed a greater tumour burden than the control-treated cells (Fig. 6D), in agreement with the *in vivo* luciferase-based imaging (Fig. 6B,C). Furthermore, the proliferation marker Ki67 was localised to the CD99-expressing tumour cells (Fig. 6D), and quantification of Ki67^+^ cells showed a significantly higher tumour burden in the mice implanted with CD99-depleted MDA-MB-231 cells (Fig. 6E).

This xenograft model identifies a complex role for CD99 in tumour progression, with CD99 expression reducing the initial accumulation of the tumour cells in the lungs, but ultimately favouring the growth of the established metastasis. This suggested that breast cancer metastases in patients might demonstrate reduced CD99 expression compared to primary tumours. We made use of published RNA-seq data obtained from the primary tumour and the matched brain metastasis of 22 breast cancer patients (Varešlija et al., 2019). A number of genes have previously been shown to be differentially expressed between the primary tumour and the brain metastases. For example, VCAM1 is downregulated in brain metastases, whereas SOX2 is upregulated (Lee et al., 2016). We confirmed this pattern of expression in this dataset and further analysis showed that CD99 mRNA was significantly downregulated in the metastases (Fig. 6F).

## DISCUSSION

The role of CD99 in leucocyte TEM during inflammation is long established (Lou et al., 2007; Muller, 2016; Schenkel et al., 2002; Watson et al., 2015). However, a role for tumour cell CD99 in TEM and metastasis is not well defined. Our results identify a functional link between CD99, CDC42 and cytoskeletal dynamics in cancer cells and demonstrate that breast cancer CD99 negatively regulates CDC42 activity and TEM. Our data showing that depletion of tumour cell CD99 increases CDC42 activity and enhances the spreading and intercalation of tumour cells into the endothelium is consistent with the work of Reymond et al. (2012), which showed that CDC42 depletion reduced both tumour cell spreading on endothelial monolayers and the metastasis of breast (MDA-MB-231) and prostate cancer (PC3) cells *in vivo*. In this study, Rac1, RhoA and CDC42 were all required for tumour cell adhesion to the EC, but only CDC42 was required for the intercalation of tumour cells into the EC (Reymond et al., 2012). The CD99 molecule has previously been linked to Rho GTPase activity, actin dynamics and growth factor signalling in breast cancer; EGFR stimulates RhoA and Rac1 activity and actin reorganization, and these pathways are antagonised by CD99-mediated signalling (Lee et al., 2020). In addition to this role in regulating growth factor responses, our data show that by negatively regulating CDC42 activity and actin reorganisation, tumour cell CD99 also modulates the TEM/metastasis of breast cancer. To our knowledge, CD99 has not been linked to Rho GTPase activity in other cell types, but in Ewing's sarcoma CD99 engagement is linked to actin reorganisation (Cerisano et al., 2004), and loss of CD99 results in neurite outgrowth, a process shown in other cell types to require cytoskeletal rearrangements and Rho GTPases, including CDC42 (Ahmed et al., 2006; Pertz et al., 2008; Xiang et al., 2016).

The CD99 molecule acts downstream of leucocyte-EC adhesion events where it plays a critical role in endothelial invasion (Schenkel et al., 2002). That CD99 functions at a relatively late stage of TEM likely explains why statistically significant effects of CD99 depletion were observed at later time points in the TEM assays employed here. Adhesion of leucocytes to the EC is CD99 independent (Schenkel et al., 2002), whereas for MDA-MB-231 tumour cells, both blocking antibodies and RNAi-mediated CD99 depletion significantly reduced tumour-EC adhesion. Differential activity of CD99 in tumour cells and healthy primary leucocytes likely reflects the number and nature of the different cell surface molecules implicated in the adhesion/TEM pathways and the relative expression of these molecules by the migrating cell type (Muller, 2016).

In the EC, the cytoplasmic tail of CD99 associates with a complex of sAC, PKA and ezrin (Watson et al., 2015). Interestingly, CDC42 is activated by PKA (Chahdi et al., 2005; Feoktistov et al., 2000), suggesting a role for CD99-associated sAC and PKA in CDC42 regulation. Furthermore, actin dynamics and cell migration are regulated by PKA and recently the PKA signalling pathway has been found to be disrupted in metastatic breast cancer (Howe, 2004; Paul et al., 2020). Increased CDC42 activity following CD99 depletion might result from enhanced CDC42 GEF activity and/or the reduced activity of the negative regulators, namely GAPs and GDIs. One of the most characterised CDC42 GEFs, β-PIX (also known as ARHGEF7), is phosphorylated and activated by PKA (Chahdi et al., 2005). Currently, the importance of CD99-mediated PKA activity in TEM has only been demonstrated for ECs, in which it triggers mobilisation of CD99 from the LBRC (Muller, 2016; Watson et al., 2015). Furthermore, ∼80 Rho GTPase GEFs have been identified and ∼50% of these are reported to regulate CDC42 (Lawson and Ridley, 2018). As such, the GEF(s) linking CD99 to CDC42 activity remains to be identified. Interestingly, a β-Pix-CDC42 pathway has recently been identified to regulate perineural invasion in pancreatic cancer (Chernichenko et al., 2020). The basis of the small increases in total CDC42 detected upon CD99 depletion are unclear, but might result from the altered activity of transcription factors and/or post-translational modifications of CDC42 that occur downstream of CDC42 activation and actin reorganisation (Corry et al., 2020; Haga and Ridley, 2016; Hall, 2012; Medjkane et al., 2009; Perona et al., 1997). Importantly, the requirement for CDC42 activity in TEM and metastasis is linked to the CDC42-dependent induction of the *ITGB1* gene, with β1 integrin playing a critical role in tumour cell intercalation into the endothelium (Reymond et al., 2012). Such activity likely occurs via the G-actin-/F-actin-dependent regulation of myocardin-related transcription factors (MRTFs), important co-factors of the serum response factor (SRF); genes regulated by MRTF/SRF and actin dynamics include those encoding key functions in cytoskeletal remodelling and metastasis (Medjkane et al., 2009).

Expression studies reveal that CD99 has both positive and negative effects on human tumour progression, depending on the tumour type (Manara et al., 2018). Our data show that CD99-depleted breast cancer cells exhibit enhanced tumour progression in a metastatic xenograft model and enhanced TEM activity *in vitro*, consistent with the tumour-suppressor-like activity of CD99 found in osteosarcoma and gastric cancer, in which reduced CD99 expression is associated with increased migration and metastasis, and reduced survival (Lee et al., 2007; Manara et al., 2006). Such activity is also supported by the reduced expression of CD99 mRNA in breast cancer brain metastases compared to their matched primary tumours. It has been reported that CD99 expression is not prognostic in primary breast cancer; however, CD99-expressing tumours were present in low numbers in this study (Czapiewski et al., 2015). Antagonism of EGFR-mediated signalling by breast cancer CD99 (Lee et al., 2020) suggests that CD99 depletion might be associated with the increased growth of metastases in our model. However, we used transient siRNA transfection, and CD99 depletion does not persist over the 4-week period that our *in vivo* model was analysed. Our xenograft model only represents the later stages of haematogenous metastasis, as the tumour cells were injected directly into the circulation. How CD99-depleted tumour cells behave during the progression of the primary tumour and its intravasation and spread is unclear. Increased progression of CD99-depleted tumour cells is consistent with increased migration in the absence of CD99 and the enhanced ability of CD99-depleted cells to disrupt EC barriers. However, we cannot dissociate TEM activity in this model from events occurring after seeding into the metastatic site; tumour-stromal interactions and innate immune recognition (which remains intact in the SCID mouse model) may also be regulated by tumour cell CD99 and contribute to progression. Interestingly, tumour cell CDC42 has previously been shown to confer resistance to cytotoxic lymphocytes and, in breast cancer cells, CDC42-dependent actin remodelling at the immune synapse reduces killing by natural killer cells (Al Absi et al., 2018; Marques et al., 2008).

Although our data suggest a tumour-suppressor-like role for CD99 in cancer progression, other cancers, such as Ewing's sarcoma and glioma, demonstrate oncogene-like activity of CD99 (Manara et al., 2018; Rocchi et al., 2010; Seol et al., 2012). The documented pro- and anti-tumour activity of CD99 is mirrored by both positive and negative roles for CDC42 in tumour progression (Stengel and Zheng, 2011). Increased expression of CDC42 is observed in several cancers, including breast and colorectal cancer (Fritz et al., 1999; Gómez Del Pulgar et al., 2008), and in melanoma, lung and testicular cancer, increased expression is associated with poor outcome (Kamai et al., 2004; Liu et al., 2009; Tucci et al., 2007). However, tumour suppressor-like activity of CDC42 has also been reported in human cancer, as well as mouse models (Valentijn et al., 2005; van Hengel et al., 2008; Yang et al., 2007).

In conclusion, we show that cancer cell surface CD99 modulates tumour cell TEM and metastatic progression, and we identify CD99 as a negative regulator of CDC42 activity. We speculate that, in a primary tumour, small numbers of cells with reduced CD99 expression gain migratory and TEM activity, allowing them to enter the vasculature. For extravasation, tumour cells are less efficient at attaching to the endothelium due to reduced CD99-mediated adhesion, but they have enhanced TEM activity, allowing them to seed a metastasis. By establishing a functional link between CD99 and CDC42 in tumour TEM, we demonstrate that CD99 plays a key role in tumour progression and implicate cell surface CD99 in controlling the diverse activities regulated by CDC42.

## MATERIALS AND METHODS

### Cells and cell culture

Human umbilical vein ECs (HUVEC) were purchased from Promocell. MDA-MB-231, MCF7 and PC3 cells were purchased from the European Collection of Cell Cultures and tested periodically for mycoplasma contamination. EC lines were cultured using endothelial cell basal medium (ECBM; Promocell), supplemented with 2% fetal calf serum (FCS) (v/v), 0.4% Endothelial Cell Growth Supplement, 0.1 ng/ml epidermal growth factor (recombinant human), 1 ng/ml basic fibroblast growth factor (recombinant human), 90 μg/ml heparin and 1 μg/ml hydrocortisone. Cells were grown on 0.2% gelatin (Sigma-Aldrich) (w/v in PBS)-coated plates. HUVEC cells were grown to passage 5 or 6. MDA-MB-231 cells were cultured in 10% (v/v) FCS (Sigma-Aldrich)-supplemented RPMI-1640 (Sigma-Aldrich) and passaged every 3-5 days. PC3 cells were cultured in 10% FCS-supplemented Dulbecco's modified essential media (Sigma-Aldrich). All cell lines were incubated at 37°C under 5% CO_2_.

### Flow cytometry

Cultured cells were PBS washed and trypsinised with 1× Acutase (Gibco). Cells were washed in ice-cold PBS followed by centrifugation at 300 ***g*** for 5 min. After repeated washing in PBS, cells were resuspended in 100 μl fluorescence-activated cell sorting buffer (PBS, 2% FCS and 0.09% NaN_3_) and stained with fluorophore-conjugated antibodies (CD99-APC, HCD99 12E7, BioLegend) and relevant isotype control antibodies at 10^6^ cells per 100 μl staining buffer for 30 min at room temperature. Stained cells were washed and fixed in Cytofix Fixation buffer (BD Biosciences) before analysis using a LSRII flow cytometer (BD Biosciences). For the siRNA deconvolution experiments, CD99 expression was analysed by imaging cytometry using a Nucleocounter NC-3000 (Chemometec) following live cell staining with CD99-APC (HCD99; BioLegend) as described above.

### Tumour-EC adhesion assay

EC were seeded at a density of 10^4^/well of a 96-well plate (Corning) and incubated for 24-48 h or until confluent monolayers were present in each well. MDA-MB-231 cells were labelled with Cell Tracker Green (CTG) for 30 min in serum free RPMI (SFM-RPMI) medium at 37°C. MDA-MB-231 cells were washed in SFM RPMI once before being seeded at 10^4^ per confluent EC monolayer. Adhesion assay was incubated at 37°C and MDA-MB-231 cells were allowed to adhere to the EC monolayers for 15, 30, 60 and 120 min, after which each plate was washed once in PBS, and fixed in 4% (w/v) paraformaldehyde (PFA; Sigma-Aldrich) for 10 min, and washed twice in PBS before storage at 4°C, followed by imaging using an Incucyte Zoom Live Cell Imager (Essen Bioscience). Images were subjected to ImageJ analysis, and the ‘watershed’ function (www.imagej.net/Classic_Watershed) was used to distinguish between individual cells and clusters.

### RNAi

HUVEC and MDA-MB-231 were transfected with SMARTpool siRNA (Dharmacon) targeting CD99. Control siRNA was used alongside untransfected (or mock transfected) cells. Transfections were performed using Lipofectamine 200 RNAiMax (Invitrogen) transfection agent and Opti-MEM I Reduced Serum Medium, GlutaMAX Supplement (Gibco) according to manufacturer's instructions. Cells were transfected with 30 pmol siRNA in a six-well plate (2-4×10^5^ cells/well) and scaled accordingly. Briefly, for a single well of a six-well plate, 30 pmol siRNA duplexes were made in 250 μl of OptiMEM medium and incubated at room temperature for 5 min. For co-depletion of CD99 and CDC42, 15 pmol of siRNA of both CD99 and CDC42 siRNA duplexes (30 pmol total siRNA concentration) were diluted in 250 μl of OptiMEM medium and incubated at room temperature for 5 min. At the same time, 5 μl Lipofectamine was made up in 250 μl of OptiMEM and incubated at room temperature for 5 min. siRNA and Lipofectamine mix were combined within the six-well plate and gently mixed before incubating at room temperature for 20 min. Following this, OptiMEM suspended cells were added to siRNA Lipofectamine complexes at 2-4×10^5^ cells in 1 ml of OptiMEM. Cells were incubated in this mixture for 4-6 h, before transfection medium was aspirated and replaced with supplemented normal culture medium. siRNA-treated cells were incubated for 24-72 h before being used in downstream assays. The siRNA molecules used (ON-TARGETplus from Dharmacon/Horizon Discovery) were as follows: human CD99 siRNA SMARTpool, 5′-GGAUGGUGGUUUCGAUUUA-3′, 5′-CUUCAUUGCUUACCAGAAA-3′, 5′-GAACCCACCCAAACCGAUG-3′, 5′-CGUUUCAGGUGGAGAAGGA-3′; human CDC42 siRNA SMARTpool, 5′-GGAGAACCAUAUACUCUUG-3′, 5′-GAUUACGACCGCUGAGUUA-3′, 5′-GAUGACCCCUCUACUAUUG-3′, 5′-CGGAAUAUGUACCGACUGU-3′; control siRNA, ON-TARGETplus Non-targeting control pool (Dharmacon/Horizon Discovery).

### Western blotting

Cells were washed in PBS before being lysed in lysis buffer [2% SDS (Sigma-Aldrich) in PBS]. Cell lysates were boiled at 100°C for 5 min, and sonicated using Sonicator (Soniprep 150 – MSE) for 1-2 s. Protein concentration of samples was then determined using a Pierce bicinchoninic acid assay BCA Protein Assay Kit (Thermo Fisher Scientific). Cell lysates were then mixed with 2× sample buffer (100 mM Tris-HCl, 4% SDS, 20% glycerol, 0.2% bromophenol blue and 10% β-mercaptoethanol) and stored at −20°C before being loaded into 8, 10, 12 or 15% SDS page gels [8,10, 12 or 15% acrylamide, 380 mM Tris (pH 8.8), 0.1% SDS, 0.1% APS and 0.1% tetramethylethylenediamine (TEMED)] set with 4% stacking gel [4% acrylamide, 120 mM Tris (pH 6.8), 0.1% SDS, 0.1% APS and 0.1% TEMED] for electrophoresis. Samples were loaded along with SeeBlue Plus 2 pre-stained protein standard (Invitrogen) and were electrophoresed for 1 h 20 min at 120 V (12 V/cm). Following transfer, membranes were blocked and probed with primary antibodies (CD99, 12E7, BioLegend; CDC42, 11A11, Cell Signaling Technology; β actin, AC-74, Sigma-Aldrich; GAPDH, 2D9, Origene) in 1% bovine serum albumin (BSA) TBS plus 0.1% Tween 20 overnight at 4°C before washing and incubation with horseradish peroxidase-conjugated secondary antibody (1:10,000) (Cell Signaling Technology). Membranes were then developed using super enhanced chemiluminescence (ECL) (Bio-Rad) and placed in a light proof case and exposed to ECL Hyperfilm (GE Healthcare) for between 1 s and ∼10 min, depending on the strength of signal. Chemiluminescence of membranes was also detected using a ChemiDoc imager (Bio-Rad) in some instances.

### Intercalation and live cell imaging

Cancer cell TEM or intercalation was determined by live cell imaging. ECs were seeded onto 96-well plates at a density of 10^4^/well in 100 μl of medium to achieve confluent monolayers in 24-48 h. Once confluent endothelial monolayers were established, CTG-labelled cancer cells were seeded onto endothelial monolayers at a density of 10^4^/well in 50 μl of medium (total medium volume of 150 μl, including endothelial culture medium). Plates were then imaged immediately using Live Cell Imager - Incucyte Zoom. Images were taken every 5 min for 4 h using a 20× objective.

For image analysis, the green imaging channel capturing CTG-labelled MDA-MB-231 cells was subjected to thresholding using the mask function within the Incucyte analysis software. A threshold based on cell size (100-μm radius) and circularity (0.7, where 0 represents a perfect circle) was created to discriminate between MDA cells that had not undergone TEM (i.e. rounded) and those which had undergone TEM. Cell number was then calculated for each individual image captured using this mask. To calculate the percentage of cells that had undergone intercalation and spreading, the remaining rounded cells that had not undergone spreading were subtracted from the initial images at time zero to determine the number of cells that had undergone spreading. An example of the analysis mask used is shown in Fig. S6.

### Transwell migration assay

Twenty-four-well Thincert 3-μm or 5-μm pore diameter transparent transwell filters (Greiner Bio-One Ltd) were coated with 0.2% (w/v) gelatin, and HUVEC were seeded at a density of 2×10^4^ cells/insert. Cells were seeded in 300 μl ECBM medium, with 500 μl in the lower chamber of the transwell insert. ECs were grown for 24-48 h to allow formation of confluent monolayers before 2×10^4^ MDA-MB-231 cancer cells were seeded to the upper chamber. MDA-MB-231 cells (2×10^5^/ml) were CTG labelled before seeding to confluent EC monolayers in 1:1 ECMB:RPMI medium. MDA-MB-231 cell migration was halted at 18 h by fixation in 4% PFA for 10 min, followed by washing twice in 1× PBS (250 μl for upper chamber and 500 μl for lower chamber). Upper chambers of transwells were then scraped using cotton wool buds to remove cells on the upper layer of the transwell insert, leaving cells that had migrated to the underside of the membrane intact. Transwells were then washed twice in PBS. Migrated cells were then imaged using an EVOS microscope (Thermo Fisher Scientific).

### Real-time cell analyser analysis using xCELLigence

Real-time monitoring of cellular proliferation, spreading and migration were measured using an electrical impedance-based assay using an xCELLigence real-time cell analyser (RTCA) DP instrument (ACEA Biosciences, Inc.). For assessment of spreading and migration, 5×10^3^ MDA-MB-231 cells siRNA reverse transfected (48 h post transfection) were seeded to E-plates (ACEA Biosciences, Inc.) and analysed using an xCELLigence RTCA DP instrument. Impedance was measured every 15 min for 24 h. For real-time monitoring of cellular migration, cell invasion/migration (CIM)-plate 16 inserts (ACEA Biosciences) were used when MDA-MB-231 cells were plated in serum-free medium to the upper chamber and the lower chamber was loaded with 10% FCS-containing complete growth medium as a chemoattractant. Impedance was measured every 15 min for 24 h. Data from each time point were exported and compiled in Excel from a minimum of three biological replicates for comparison of siRNA-treated cells.

For monitoring of endothelial barrier disruption by cancer cells, E-plates were coated, prior to plating ECs, with 10 μg/ml human fibronectin (Sigma-Aldrich) for 2 h at room temperature under a laminar flow hood. Wells were then rinsed twice in PBS and air dried before seeding of ECs. ECs were seeded at 10^4^ in 100 μl/well of an E-plate and plates were loaded into an xCELLigence RTCA DP instrument. ECs were grown to confluency for 24-48 h before the addition of cancer cells. MDA-MB-231 cells (10^4^) in 50 μl were loaded into wells containing EC monolayers, and impedance was measured every 15 min for 24 h. Data were analysed using fold change in barrier integrity upon seeding of cancer cells to EC monolayers compared to HUVEC monolayers alone.

### Extracellular matrix adhesion, immunofluorescence and actin quantification

ViewPlate 96-well black plates (PerkinElmer) were coated with either 0.2% w/v gelatin, fibronectin (10 μg/ml in PBS) (ProSpec*-*Tany TechnoGene, Ltd) or collagen type 1 (10 μg/ml in 0.1 M acetic acid; Merck Millipore). Plates were incubated at 37°C for 2 h before aspirating excess ECM coating and rinsing twice with PBS, and air drying for 30 min at room temperature. A density of 10^4^ cells/well of siRNA-treated MDA-231 cells were seeded to a coated ViewPlate 96-well plate and allowed to adhere for 30 min. Unbound cells were then washed away in PBS and fixed in 4% PFA for 10 min at room temperature. Cells were then permeabilised in 0.2% Triton X-100 and blocked for 1 h in 3% BSA PBS before staining using Texas Red phalloidin [Thermo Fisher Scientific (1:200)] and Hoechst (1:10,000) in PBLEC staining buffer (0.1% BSA, 1% Tween 20, 0.05% NaN_3_, 1 mM CaCl_2_, 1 mM MgCl_2_ and 0.1 mM MaCl_2_) at room temperature for 2-4 h. Following this, cells were rinsed twice in PBS before subsequent imaging was carried out using an Operetta HTS imager (PerkinElmer). Image analysis was performed using Columbus software. Three-dimensional actin distribution plots were generated using ImageJ ‘3D surface plot’ analysis.

For confocal imaging of actin, siRNA-treated MDA cells were seeded to collagen type 1 (10 μg/ml in 0.1 M acetic acid; Merck Millipore)- or fibronectin (10 μg/ml in PBS; ProSpec*-*Tany TechnoGene, Ltd)-coated eight-well chamber slides (Corning) and allowed to adhere for 30-45 min before being gently washed in PBS and fixed in 4% PFA for 10 min. Cells were then gently washed twice in PBS. Cells were permeabilised using 0.2% Triton X-100 (Sigma-Aldrich) for 5 min. Cells were blocked in 5% horse serum in 1× PBS for 1 h prior to staining using Texas Red phalloidin [Thermo Fisher Scientific (1:200)] for 2-4 h at room temperature. Chamber slides were washed twice in PBS before mounting using DAPI containing Fluoromount-G (SouthernBiotech) (for 24 h at room temperature away from light). Cells were analysed using a confocal laser scanning microscope LSM 700 (Leica). For analysis and quantification of the peripheral actin cytoskeleton, actin intensity was measured along a 15-μm intersection radially from the centre of an individual cell to the cell periphery using ImageJ. A total of 12 cells were analysed from three random fields of view across three independent experiments (total of 36 cells analysed). Peak values of arbitrary units of intensity at the cell periphery were then normalised to the cytoplasmic actin signal to give peripheral actin intensities.

### *In vivo* metastasis model

Female CB17-SCID mice (aged 6-8 weeks) were purchased from Charles River Laboratories. All *in vivo* procedures were performed in accordance with the Animals (Scientific Procedures) Act 1986 Amendment Regulations 2012 following ethical review by the University of Leeds Animal Welfare and Ethical Review Committee (Home Office UK PPL No. 70/7544). Seventy-two hours following siRNA knockdown, luciferase expressing MDA-MB-231 cells were detached using trypsin and resuspended in RPMI-supplemented medium. Cells were strained using 70-μm cells strainers (Thermo Fisher Scientific) to remove aggregates of cells, and were pelleted before resuspending at 5×10^6^/ml in PBS. siRNA-transfected cells for injection were analysed for viability using live/dead staining and flow cytometry (Fig. S2A), and 5×10^5^ cells/mouse were injected into the main tail vein of mice; injections were performed under the approved UK Home Office project licence (No. 70/7544). For imaging, D-luciferin substrate (Sigma-Aldrich) was injected intraperitoneally into mice (2 μg/mouse), and mice were anaesthetised with isoflurane and scanned using an *In Vivo* Imaging System (IVIS) Spectrum imaging unit (PerkinElmer). Bioluminescent imaging of mice was carried out at 6 h, 24 h, 48 h and 72 h post injection, and then weekly for 4 weeks. For analysis of tumours, formalin-fixed paraffin-embedded lungs were sectioned (6 µm), de-waxed and rehydrated prior to staining with hematoxylin and eosin or processing for immunofluorescence microscopy following heat-induced antigen retrieval [10 mM sodium citrate (pH 6.0) and 0.1% Tween 20]. Sections were stained overnight with rabbit anti-Ki67 AB9260 (Millipore) and mouse anti-CD99 (12E7, BioLegend). Images were captured on an EVOS FL microscope (Life Technologies) and protein expression quantified in ImageJ as described previously (Williams et al., 2017). Briefly, equivalent captured images were subjected to constant thresholding prior to intensity density measurement. These values were used to calculate corrected fluorescence intensity and percentage area covered.

### CDC42 pulldown

GTP-bound CDC42 was assessed using a CDC42 Activation Assay Biochem Kit (Cytoskeleton, Inc.). Cells were grown to confluency in T75 flasks, then detached, washed in appropriate medium and counted. Cell density was adjusted so that equal numbers of cells were used per condition. An aliquot of 3-5×10^6^ cells were reseeded to a 0.2% gelatin-coated T75 flask at equal densities (per condition of siRNA treatment), and left to adhere for 30 min for HUVEC and 4 h for MDA-MB-231 cells. Following this, cells were placed on ice and the medium was removed. Cells were gently washed in ice-cold PBS, which was quickly aspirated. Cells were then lysed in 500 μl ice-cold lysis buffer (provided in kit) and lysates were collected using a cell scraper and placed in 1.5 ml Eppendorf tubes on ice. Protein (300-800 μg, as per the Cdc42 Activation Assay Biochem Kit protocol) was added to 20 μl (20 μg) of p21 activated kinase 1 protein (PAK1) – p21 binding domain (PBD) coated beads that were incubated on a rotator at 4°C for 1 h. PAK1-PBD beads and lysate were then pelleted by centrifugation at 3000-5000* **g*** at 4°C for 1 min. Pelleted beads were then mixed with 20 μl 2× Laemmli sample buffer and thoroughly resuspended. Samples were boiled at 100°C for 2 min before analysis by SDS-PAGE using 15% gels. Input control lysates, which had not been subject to immunoprecipitation with PAK1-PDB beads, were electrophoresed alongside pulldown samples to determine total CDC42 protein expression.

### Cell proliferation *in vitro*

Cell proliferation was determined using a Crystal Violet staining assay (Feoktistova et al., 2016). Briefly, 10^4^ cells were cultured for 24, 48, 72 and 96 h under normal culture conditions following siRNA transfection. Following culture for the indicated time periods, cells were washed, fixed and stained in 1 ml of Crystal Violet staining solution [0.5% Crystal Violet powder (Sigma-Aldrich), 20% Methanol] for 20 min at room temperature with gentle agitation. Stained cells were solubilised in 1 ml 100% methanol/well and incubated with gentle agitation for 20 min at room temperature with lids on the plate. The optical density of each well was measured at 570 nm (OD_570_) using a plate reader. Average OD_570_ of control empty wells was subtracted from OD_570_ of wells containing cells.

### Scratch wound assay

GFP-expressing MDA-MB-231 cells were seeded at a density of 2×10^4^/well in a 96-well plate in 100 μl of 10% FCS-supplemented RPMI medium and grown to confluency over 24-48 h. Once confluent, cells were serum starved for 2 h prior to ‘wounding’. Culture medium was removed using an aspirator and replaced with serum-free RPMI medium (supplemented with 1% BSA). Following serum starvation, scratch wounds were made using the ‘Incucyte wound maker implement’. Once scratched, medium was gently removed by aspirator and monolayers were gently washed with PBS before being replaced with supplemented RPMI medium. The assay was imaged after 18-24 h to assess the closure of the wound. Images were analysed using ImageJ ‘MRI_Wound_Healing_Tool-1’ plug-in.

### Patient gene expression data

The RNA-seq data generated by Varešlija et al. (2019) was downloaded and analysed for the expression of *VCAM1*, *SOX2* and *CD99* genes in the matched primary and brain tumour metastases from 22 breast cancer patients using paired non-parametric statistical testing.

### Statistical analysis

Statistical analysis was performed with data generated from *n*≥3 biological replicates using GraphPad Prism7 software. Error bars represent s.e or s.d. from the mean as described in the figure legends. The different statistical tests used for individual experiments are also described in the figure legends. Results were deemed significant if *P*<0.05 and were denoted as: *P<0.05, **P<0.005 and ***P<0.0005.

## Supplementary Material

Supplementary information

Reviewer comments
