# Supplementary material for: Tumour cell CD99 regulates transendothelial migration via CDC42 and actin remodelling
Source: J Cell Sci. 2021 Aug 10;134(15):jcs240135. doi: 10.1242/jcs.240135 (PMC8403985; doi:10.1242/jcs.240135)
Supplement: Supplementary information [file joces-134-240135-s1.pdf]

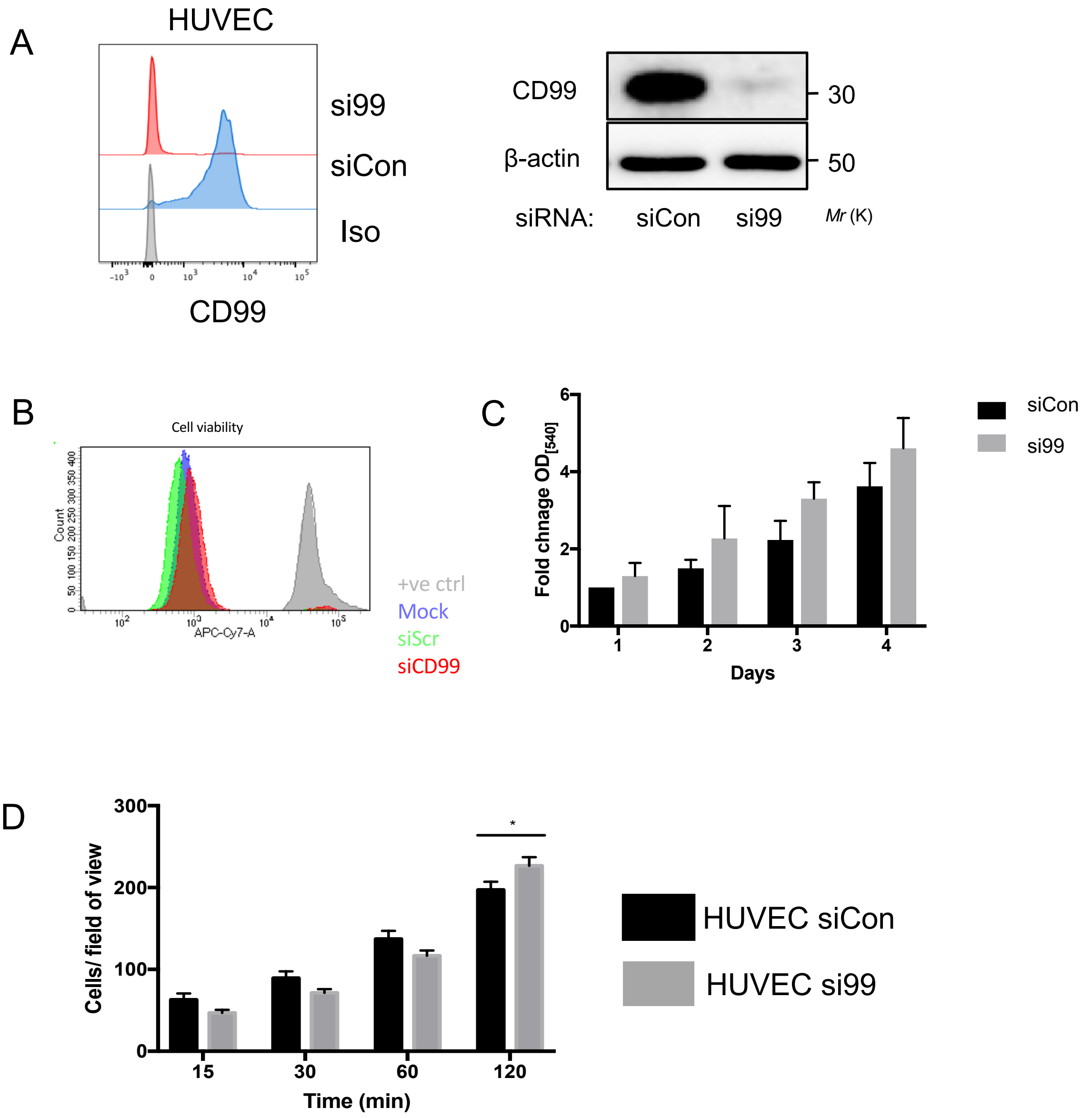


Journal of Cell Science • Supplementary information

**Fig. S1.**

**A** siRNA mediated knockdown of CD99 in HUVEC determined by flow cytometry and western blotting; HUVEC were transfected with siRNA targeting CD99 (si99) or a control siRNA (siCon) for 72h before analysis of cell surface CD99 by flow cytometry. Cells were stained with anti-CD99 antibody or an isotype control (iso). Blots were probed with an anti-CD99 antibody and an anti-β actin antibody as shown. Data are representative of three independent replicates

**B** Cell viability of mock, control (siCon) and CD99 (si99) siRNA transfected MDA-MB-231 cells was assessed using cell viability staining. Treated cells were stained with Zombie dye (from BioLegend) to discriminate live and dead cells; cells fixed in 4% PFA were included as a positive control for dead cells.

**C** Proliferation of MDA-MB-231 after CD99 depletion. MDA-MB-231 cells treated with control or CD99 siRNA (si99) were seeded to 24 well tissue culture dishes at the same density. Plates were left for 1, 2, 3 and 4 days before fixing in 4% PFA and staining with Crystal Violet. Stained cells were solubilised in 100% methanol and spectrophotometry performed using a plate reader. Results were normalised to stained empty wells and background subtracted. Data are normalised to siCon treated cells at day 1 of the assay and show three replicates. Error bars indicate standard deviation. Proliferation was not statistically different between the samples based on an unpaired Student's t test.

**D** Adhesion of MDA-MB-231 cells to CD99 depleted HUVECs. HUVEC cells were transfected with control (siScr) or CD99 siRNA (si99) for 72h and grown to confluent monolayers before seeding of CTG-labelled MDA-MB-231 and left to adhere for the indicated time points. Unbound cells were washed away with PBS before fixation in 4% PFA and quantification of bound cells. Error bars indicate standard Error. Statistical analysis was performed using Student’s t-test. *$p<0.05$.

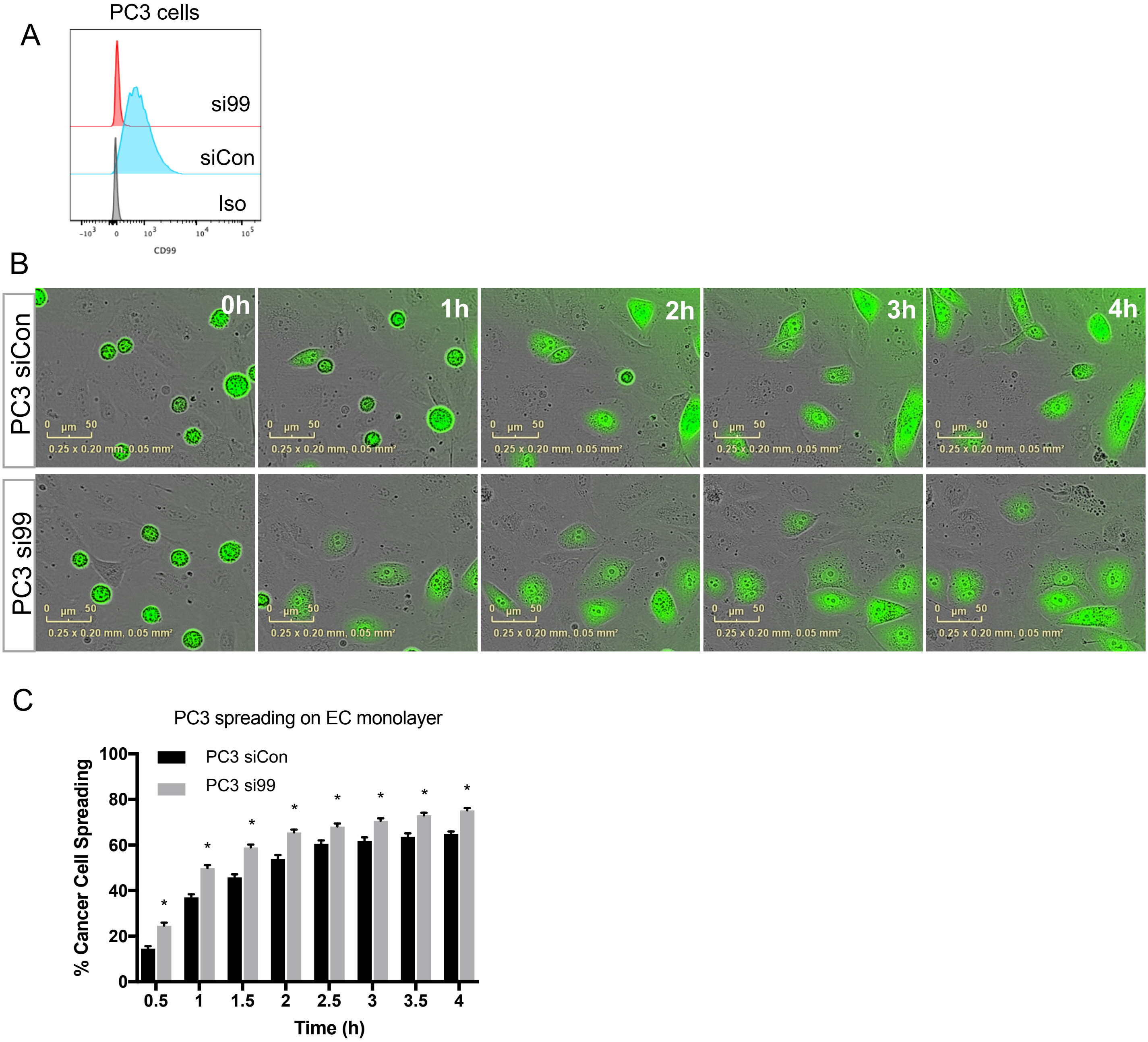


**Fig. S2. A** Prostate cancer PC3 cells were transfected with siRNA targeting CD99 (si99) or a control siRNA (siCon) for 72h before analysis of cell surface CD99 by flow cytometry. Cells were stained with anti-CD99 antibody or an isotype control (iso). Data are representative of three independent replicates. **B** PC3 transfected with CD99 siRNA (si99) or control siRNA (siCon) were labelled with Cell Tracker Green before seeding on top of confluent HUVEC monolayers and imaging for 4 hours using an Incucyte live cell imager. Scale bar: 50 µm.

**C** Quantification of data in panel B, showing intercalation of PC3 cells (treated with si99 or siCon as indicated) as a percentage of total PC3 cells. Data is representative of n=3 independent replicates. Error bars indicate standard error. Statistical analysis was performed using student t-test. *$p<0.05$.

Journal of Cell Science • Supplementary information

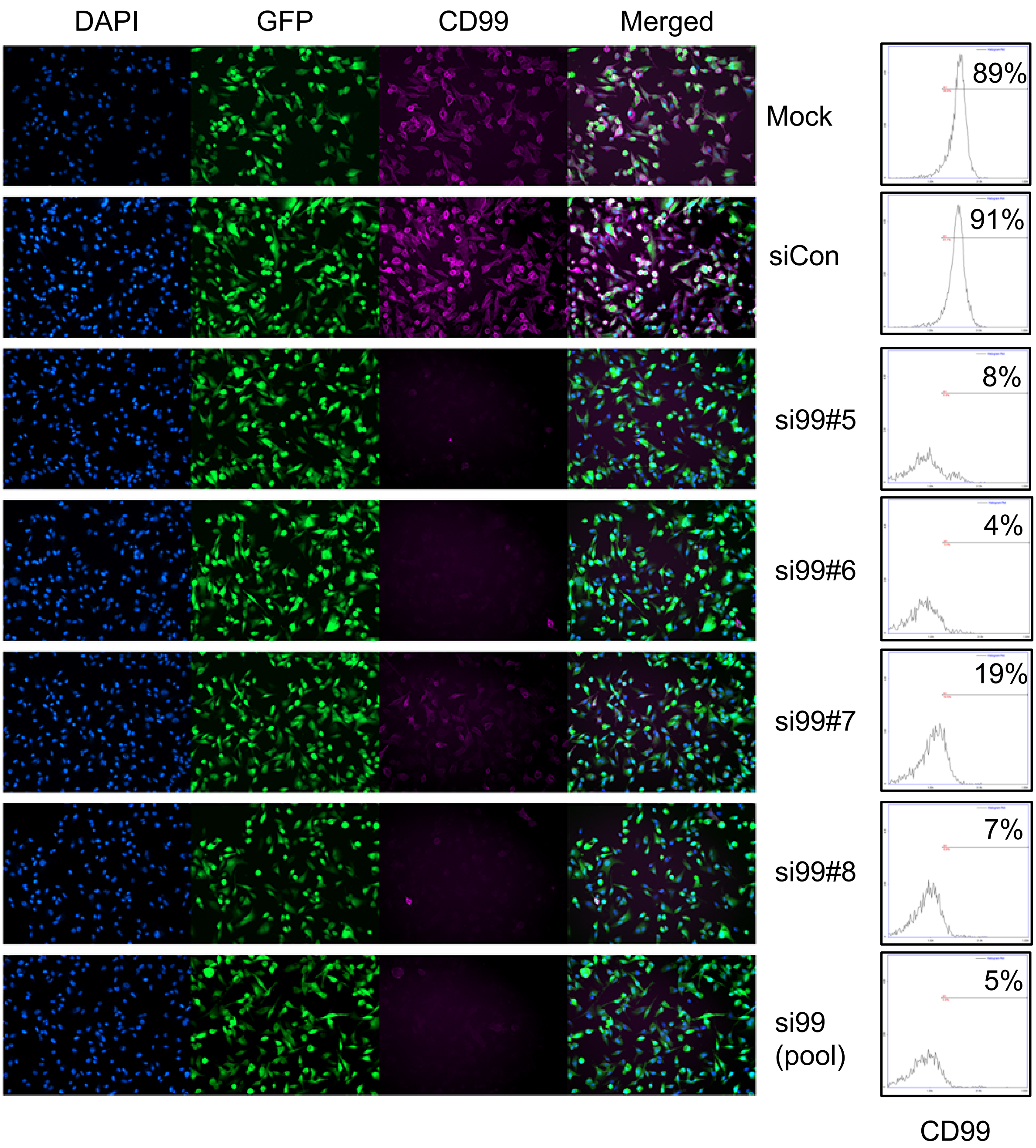


**Fig. S3.** Deconvoluted CD99 siRNA pool depletes CD99 expression. GFP expressing MDA-MB-231 cells were transfected with the CD99 siRNA pool (si99 pool) or indicated single siRNA oligos targeting CD99 (si99#5-8), control siRNA (siCon) or transfection reagents alone (Mock). 72h post transfection, knockdown efficiency was analysed by immunofluorescence using CD99 specific antibodies. Quantification indicates CD99 expression following treatment as determined by imaging cytometry using a Nucleocounter NC-3000. Data is representative of n=3 independent replicates.

Journal of Cell Science • Supplementary information

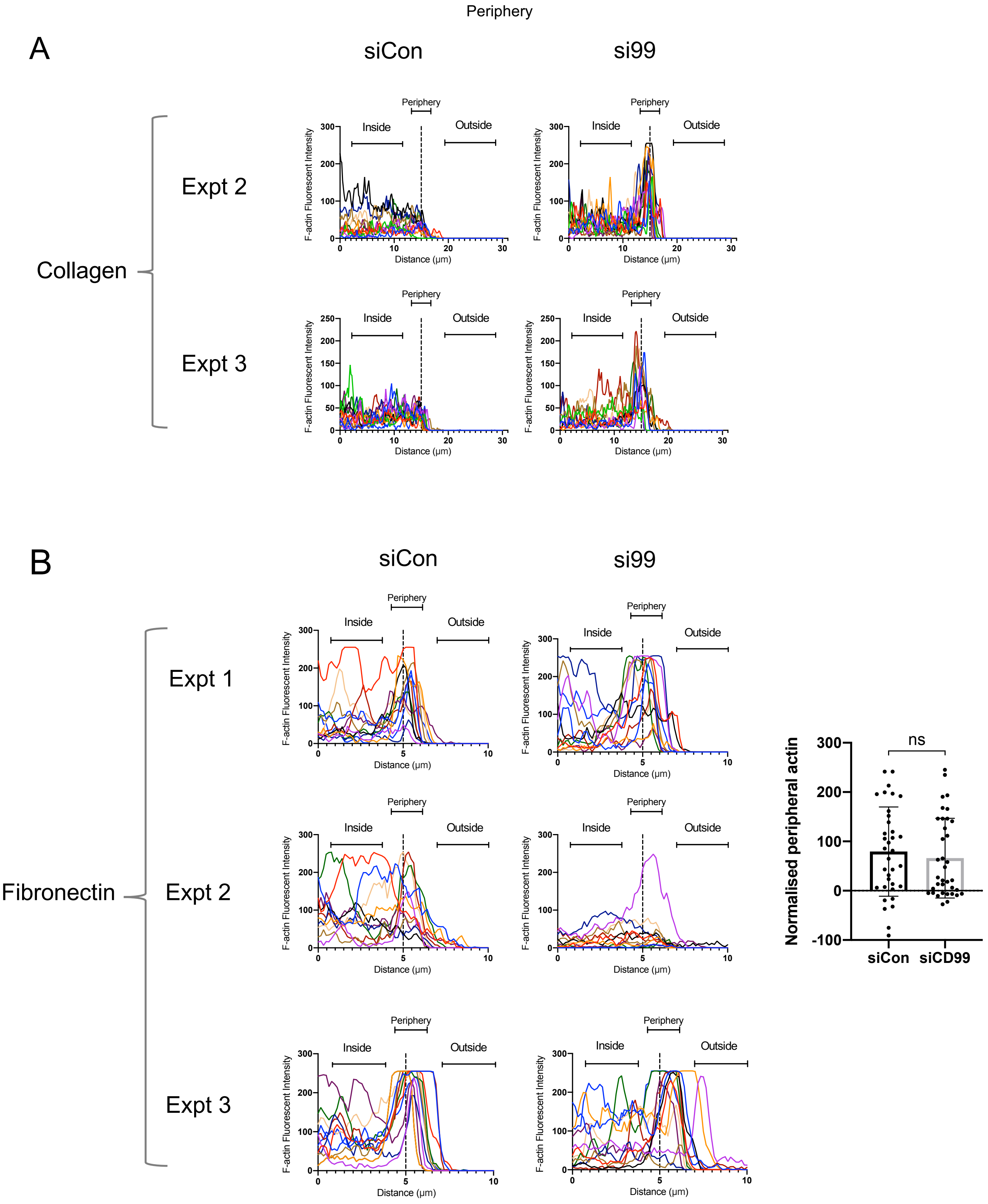


Journal of Cell Science • Supplementary information

**Fig. S4.** Quantification of peripheral actin in MDA-MB-231 cells adhering to ECM components. siRNA treated cells were imaged using LSM 700 confocal microscopy and actin intensity analysed using ImageJ. **A** Trace plots of MDA-MB-231 cells seeded to collagen indicate actin intensity inside, at the cell periphery and outside the cell (as indicated) for 12 individual cells (4 cells each from 3 random fields of view) overlayed in each graph. Data from independent experiments is shown and results from a third experiment are shown in Fig. 5 of the main manuscript, alongside the quantification of data from all experiments using collagen. **B** As in panel A, except MDA-MB-231 cells were seeded onto fibronectin coated coverslips and actin intensity trace plots generated as described in A. Overlays show 3 independent experiments. The bar graph indicates values of actin intensity at the cell periphery normalised to the cytoplasmic actin of 36 cells from the 3 independent experiments shown in trace plots. Data was analysed using an unpaired Student's t test; ns, not significant.

Journal of Cell Science • Supplementary information

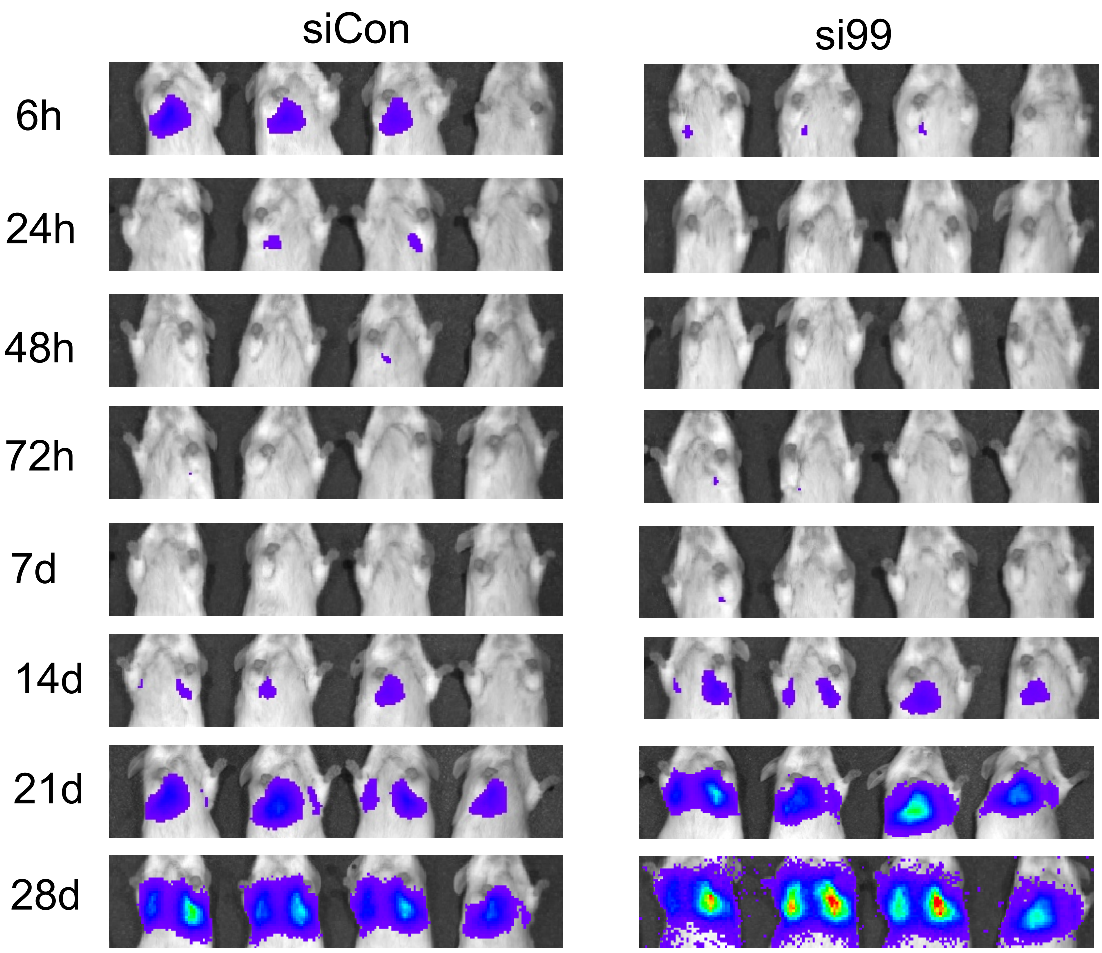


**Fig. S5.** Luciferase expressing MDA-MB-231 cells were transfected with control siRNA (siCon) or CD99 siRNA (si99). 72 h following siRNA knockdown, 5x10$^5$ cells/mouse were injected into the main tail vein of female CB17-SCID mice (aged 6-8 weeks). For imaging, D-luciferin substrate was injected and anaesthetised mice scanned using IVIS Spectrum Imaging at indicated time points. n=4 mice per group.

Journal of Cell Science • Supplementary information

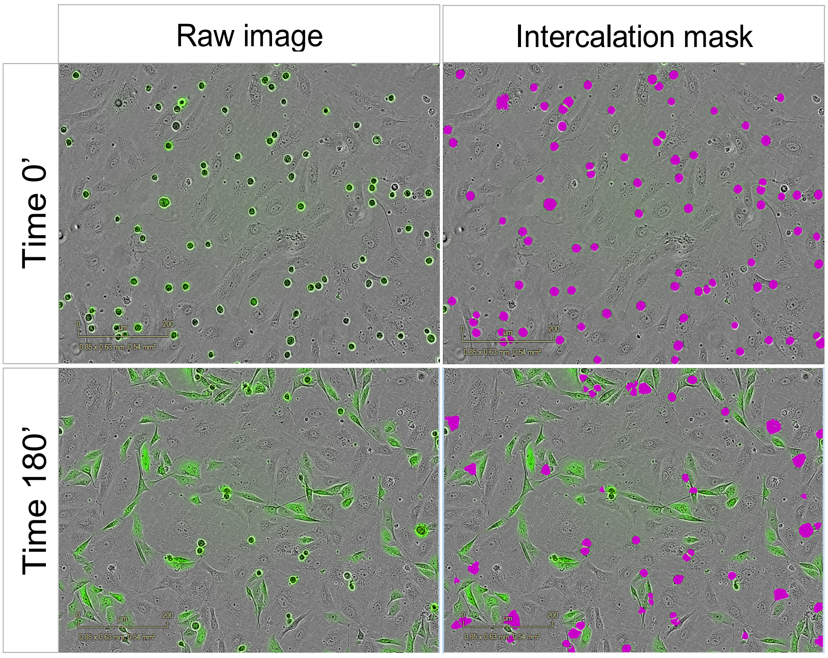


**Fig. S6.** Image analysis of MDA-231 intercalation. Example merged images of CTG labelled MDA-231 cells at 0 and 180 minutes seeded to confluent HUVEC monolayers. Intercalation mask (purple mask) to discriminate between MDA-MB-231 cells which have undergone TEM was created using the Incucyte basic analyser software function and was used to quantify the number of MDA cells which had undergone TEM. Scale bar: 200μm.
