# Supplementary material for: Tumour cell CD99 regulates transendothelial migration via CDC42 and actin remodelling
Source: J Cell Sci. 2021 Aug 10;134(15):jcs240135. doi: 10.1242/jcs.240135 (PMC8403985; doi:10.1242/jcs.240135)
Supplement: Reviewer comments [file joces-134-240135_review_history.pdf]

Tumour cell CD99 regulates transendothelial migration via CDC42 and actin remodelling

Aarren J. Mannion, Adam F. Odell, Alison Taylor, Pamela F. Jones and Graham P. Cook
DOI: 10.1242/jcs.240135

Editor: Daniel Billadeau

Review timeline

Original submission:	4 October 2019
Editorial decision:	28 October 2019
First revision received:	21 June 2021
Editorial decision:	24 June 2021
Second revision received:	2 July 2021
Accepted:	6 July 2021

Original submission

First decision letter

MS ID#: JOCES/2019/240135

MS TITLE: CD99 regulates cancer cell transendothelial migration and endothelial cell function via CDC42 and actin remodelling

AUTHORS: Aarren J Mannion, Adam F Odell, Alison Taylor, Pamela F Jones, and Graham P Cook
ARTICLE TYPE: Research Article

We have now reached a decision on the above manuscript.

To see the reviewers' reports and a copy of this decision letter, please go to: https://submit-jcs.biologists.org and click on the 'Manuscripts with Decisions' queue in the Author Area. (Corresponding author only has access to reviews.)

As you will see, the reviewers raise a number of substantial criticisms that prevent me from accepting the paper at this stage. They suggest, however, that a revised version might prove acceptable, if you can address their concerns. If you think that you can deal satisfactorily with the criticisms on revision, I would be pleased to see a revised manuscript. We would then return it to the reviewers.

Several issues were raised that need to be paid close attention to in the revision. These include rewriting of the Abstract for clarity, and the Introduction to introduce the problem in the context of the cell models being used and providing the mose relevant information regarding known functions of CD99. The angiogenesis experiments appear out of place with the rest of the manuscript and it is felt that the analysis of CD99 in endothelial cells is far too preliminary. Both should be removed from the manuscript.

Both reviewers also indicate that more mechanistic insight into how CD99 regulates Cdc42 expression/activity (what GEF is involved? Can the authors show increased Cdc42 activity following perturbations of CD99?) need to be performed. In addition, a key experiment would be to show that loss of Cdc42 impairs TEM in cells where CD99 is also depleted. They also suggest that other Rho family members should be examined such as Rac1.

© 2021. Published by The Company of Biologists under the terms of the Creative Commons Attribution License (https://creativecommons.org/licenses/by/4.0/).

Lastly, quantification of image and immunoblot data are required as is the presentation of more representative examples of IF images. Also biological replicates and appropriate statistics need to be presented.

Please ensure that you clearly highlight all changes made in the revised manuscript. Please avoid using 'Tracked changes' in Word files as these are lost in PDF conversion.

I should be grateful if you would also provide a point-by-point response detailing how you have dealt with the points raised by the reviewers in the 'Response to Reviewers' box. Please attend to all of the reviewers' comments. If you do not agree with any of their criticisms or suggestions please explain clearly why this is so.

Reviewer 1

Advance summary and potential significance to field

how cancer cells cross the vasculature is crucial to know. This paper attempts to show that CD99 plays an important role here.
For tumor TEM, this is a new player, but not for leukocyte TEM. The mechanism the authors provide is supported with limited data points.

Comments for the author

The authors present on how tumor cells manage to migrate through the endothelial barrier and implicate CD99 and CDC42 in this process. CD99 expressed by the tumor cell was shown to positively regulate the migration across endothelium. The loss of CD99 led to the remodeling of the actin cytoskeleton of the tumor cell. Remarkably, loss of CD99 in endothelial cells reduced its barrier function and yet reduced tumor cell transmigration. They furthermore state that this promotes EC angiogenesis through activation of Cdc42.
The paper is somewhat puzzling and feels more like a collection of experiments rather than a solid concise story. The fact that CD99 is involved in TEM is not novel. Several groups have reported on this. The mechanistic part is limited.

I have listed my other concerns below.
• The second part of the abstract becomes very unstructured and needs to be rewritten. It is not clear if they describe CD99 from the EC or tumor cell.
• Please re-read the introduction.
• Some grammar mistakes and LRBC = compartment, not complex. Also, remove REF.
• A difference of only 10% due to lack of CD99 is not very convincing (Fig 2B).
• It is not clear to me why the authors looked at CDC42. Spreading is clearly a Rac phenotype (also CDC42). Why not looking at that GTPase?
• I would suggest restructuring the paper. Why is the part with angiogenesis included? I would like to have seen some more mechanistic evidence on how CD99 may control CDC42 activity and not one of the other closely related GTPases. And is there a potential GEF involved?

Reviewer 2

Advance summary and potential significance to field

The study "CD99 regulates cancer cell transendothelial migration..." by Mannion et al investigates molecular mechanisms that regulate a critical step in cancer metastasis: transendothelial migration required for cancer cell extravasation and colonization of secondary sites. Understanding the mechanisms that control the ability of cancer cells to invade and metastasise is critical for the development of new therapeutic strategies and monitoring/diagnostic tools that may eventually benefit patients. In addition, it will inform of the fundamental biology underpinning cell migration that may be useful in other disciplines (immunology development, etc).

© 2021. Published by The Company of Biologists under the terms of the Creative Commons Attribution License (https://creativecommons.org/licenses/by/4.0/).

The work presented here is significant because it identifies a new regulator of cancer metastasis and provides clues about the molecular mechanisms downstream of CD99.
In this study, the authors show that the transmembrane receptor CD99 can modulate the ability of cancer cells to interact with the endothelia and cross the endothelial barrier, ultimately affecting the ability of cancer cells to metastasise. The paper shows that CD99 can operate by modulating Cdc42 levels and activity, which are required for cancer cells to rearrange their cytoskeleton change shape and cross the endothelial barrier. In addition, the authors show an additional role of endothelial CD99 in modulating the endothelial barrier function that also contributes to transendothelial migration.
Overall, the study presents new data on the mechanisms of transendothelial migration and the role of CD99 that is very interesting and has potential. The manuscript is well written and in most cases very clear and straight forward. While the data are exciting, as presented in its current format, the work lacks important conceptual and technical details that are need in order to support the author’s conclusions.

Comments for the author

Briefly, this study uses in vitro assays that show that CD99 promotes cancer cell interaction to endothelia monolayers, but inhibits cancer cell spreading and transmigration. Simple molecular characterisation suggests that CD99 operates via Cdc42 regulation. In vivo (tail-vein injections) confirms a role for cancer cell CD99 in metastasis.
Overall, the study presents new data on the mechanisms of transendothelial migration and the role of CD99 that is very interesting and has potential. The manuscript is well written and in most cases very clear and straight forward.

However, there are several concerns that need to be addressed before publication.

Major comments:
1) The study shows CD99 expression in different breast cancer lines and then uses the metastatic cell line MDA-MB-231 to experimentally demonstrate a role of CD99 in TEM. It is not clear why the authors chose this specific cell line if they were planning to perform gain-of-function assays (i.e. gaining metastatic capabilities) – it would have made more sense to use a low metastastic cell line (e.g. MCF7).

a) First of all, quantification of the differential expression of CD99 in these systems is required, as the FACS analysis (Fig 1a) does not illustrate well whether there are differences in expression.

b) It appears that MCF7 cells express more CD99, which is in line with the proposed role presented in this study. However, this needs to be confirmed appropriately. It will be good to compare expression levels of CD99 between high-metastatic (MDA-231) and low-metastatic cell lines (e.g. MCF7) against normal epithelial cells (e.g. MCF10A).

c) If that is the case, it would be appropriate to perform perturbation assays with MCF7 cells to test whether blocking CD99 function leads to the acquisition of TEM and metastatic capabilities.

d) The rationale for the inclusion of MDA-BrM in the analysis is not clear. In particular since it appears to have more CD99 expression that the parental cell line, which argues against a critical role in their metastatic potential. In addition, how do the authors reconcile this data with the lower CD99 expression observed in brain mets vs primary tumours (Fig 4f)?

2) The authors focus in a potential role of Cdc42 downstream of CD99 to explain some of their results. However, modulation of other Rho GTPases (i.e. RhoA Rac1) may yield similar results.

a) It is critical to assess that CD99 does not affect other Rho GTPases.

b) In addition, the characterisation of the role of CD99 as a potential modulator of cytoskeleton and cell shape is very poor. Changes in F-actin are barely evident and unbiased quantification of F-actin features is required. Is this phenotype only observable in cells seeded on collagen?

© 2021. Published by The Company of Biologists under the terms of the Creative Commons Attribution License (https://creativecommons.org/licenses/by/4.0/).

c) The role of Cdc42 downstream of CD99 needs to be confirmed experimentally.
For example, does Cdc42 suppression diminish the effects of loss of CD99 in TEM processes?

d) It is also very important to highlight that apparently CD99 affects primarily Cdc42 expression/stability, as shown by the increase of total Cdc42 levels. Analyses of Cdc42-GTP/total Cdc42 levels will provide information on whether Cdc42 is more active or there is simply more Cdc42. The authors need to discuss this fact in particular in regards to previous literature, as they suggest that downstream effectors of CD99 (i.e. sAC and PKA, page 17) have been involved in regulating Cdc42 activity – do they affect Cdc42 levels/stability? Does modulating sAC or PKA affect the cytoskeletal features/TEM induced by loss of CD99?

3) Overall, the experiments are well designed, but the study requires more rigorous analyses and description.

a) Each experiment needs to state the number of biological replicates performed.

b) Quantification of western blots and statistical analysis of band intensities is missing for all the experiments shown. All western blots should be accompanied by quantification of the bands with appropriate replicates and corresponding statistical analyses. Same occurs with FACS analysis

c) Generally speaking all the images are not very representative: exemplars in Fig 1B, 5F are not appropriate as they do not show great observable differences –
how do the authors identify individual cells vs cell clumps in the cell adhesion experiments?; Fig S3a is variably seen; bar and bar description are not provided
(or barely observable) in most of the images. Finally, metastasis (Fig 4) are hardly seen among the tissue; the authors should consider using outlining the metastatic foci from the rest of the tissue.

d) It is not clear how the authors analyse TEM and intercalation (i.e. Fig 2).
Is this all based in cell morphology/area? How do the authors determine if a cell is "spread"? Do the authors correct for cell on/between the endothelial monolayer?
Can they identify the cells that have migrated through the monolayer? For inferring TEM capabilities, it is critical that the authors use more standard assays such as TEM through endothelial monolayers in transwells and count the cells that have successfully transmigrated. Altogether, these analyses (as presented and described) are prone to bias which may affect the conclusions.

4) Key experiments lack proper controls. It is essential that the authors deconvolute the RNAi Smartpool into individual RNAi to discard off-target effects in key experiments.

5) When assessing adhesion (Figure 1c and 1f) and spreading (e.g. Figure 5b),
the authors need to discuss and provide a reasonable explanation as why in certain time points there are no significant differences between control and experimental points. For example, in Figure 1F there are no significant differences at 120 min but there are at previous time points. This suggests that possibly the efficacy of the process is not altered, just the dynamics.

6) The authors show that adhesion to plastic is not affected by CD99 depletion, but then they assess migration by wound healing in plastic. What about migration in gelatin/collagen/endothelial monolayer where they show that CD99 depletion has an effect?

7) For the in vivo assays the authors need to show that CD99 perturbation does not affect survival in circulation and arrest at lung capillaries to discard potential effects independent of TEM. IHC analysis of lungs at early time points (~2h post injection of fluorescently labelled cells) should provide that type of information. In addition, any potential effect of CD99 in cancer cell growth/survival needs to be discarded more rigorously using proliferation assays or similar. Data showing ki67 staining in vivo is not convincing. In fact, it is not clear how they assess ki67 expression vs ki67-positive cells and its relation with cancer cell growth.
8) The analyses of CD99 role in EC are essentially anecdotal without further characterisation. Analyses of cell-cell junction, cytoskeletal features, EC migration etc are required to provide information regarding CD99 function in EC and its relation to TEM.

© 2021. Published by The Company of Biologists under the terms of the Creative Commons Attribution License (https://creativecommons.org/licenses/by/4.0/).

Minor comments:

- The abstract is not very clear in describing the mechanistic findings particularly in what concerns CD99 role in cancer cells vs endothelia.

- Page 3. This sentence is confusing and needs revision, as it is not clear whether CD99 reduces migration AND tumour growth, or whether reduces migration but increases tumour growth: “[...] in particular in Ewing’s sarcoma where lower CD99 expression results in reduced migration, tumour growth and metastasis”.

- Since the authors focus on analysis of CD99 in breast and prostate cancer models, the clinical/biological background of this molecule in those settings needs to be introduced in the Background section. In addition, a clear statement describing the use the particular BC and prostate cancer models in this study needs to be reasoned.

- As explained before, the authors need to be careful in discussing the results on Cdc42, in particular references to CD99 negatively impacting Cdc42 activity. Data shows that it mainly affects protein expression/stability.

- Figure 1b: what time point is shown?

First revision

Author response to reviewers' comments

Response to reviewers

We thank the referees for their detailed reviews and for the constructive suggestions regarding improvement of the manuscript. The comments are responded to below. The manuscript itself has been extensively revised. We apologise for the length of time that this taken due to the pandemic. We have highlighted the major changes to the main text in yellow. All references used below are cited in the manuscript.

Reviewer 1 Comments for the Author:

Comment: The authors present on how tumor cells manage to migrate through the endothelial barrier and implicate CD99 and CDC42 in this process. CD99 expressed by the tumor cell was shown to positively regulate the migration across endothelium. The loss of CD99 led to the remodeling of the actin cytoskeleton of the tumor cell. Remarkably, loss of CD99 in endothelial cells reduced its barrier function and yet reduced tumor cell transmigration. They furthermore state that this promotes EC angiogenesis through activation of Cdc42.The paper is somewhat puzzling and feels more like a collection of experiments rather than a solid concise story. The fact that CD99 is involved in TEM is not novel. Several groups have reported on this. The mechanistic part is limited.

Response: we have focussed our revised manuscript on the role of CD99 in tumour cell TEM and metastasis. Apart from a single experiment analysing the role of endothelial cell (EC) CD99 in tumour cell adhesion (Supplementary Figure S1) the revised manuscript contains no analysis of CD99 function in EC.

It is true that a role for CD99 in TEM of leucocytes is well established. However, no studies have been reported on CD99 in tumour cell TEM and its importance in metastasis. Furthermore, our results showing CD99 mediated inhibition of CDC42 activity (Figure 5) and the impact on cell motility (Figure 3) and actin dynamics (Figure 4) have not been demonstrated previously, neither in tumour cells nor leukocytes.

Comment: The second part of the abstract becomes very unstructured and needs to be rewritten. It is not clear if they describe CD99 from the EC or tumor cell. Please re-read the introduction.

© 2021. Published by The Company of Biologists under the terms of the Creative Commons Attribution License (https://creativecommons.org/licenses/by/4.0/).

Some grammar mistakes and LRBC = compartment, not complex. Also, remove REF.

Response: We have removed the endothelial cell and angiogenesis data and focussed on the role of tumour cell CD99. This focus has allowed us to improve the clarity of the revised manuscript.

Comment: A difference of only 10% due to lack of CD99 is not very convincing (Fig 2B).

Response: This data remains in Fig 2B of the revised manuscript. We agree that a 10% increase in cell spreading upon CD99 depletion is relatively small. However, this is a statistically significant difference and, more importantly, this difference results in a consistently altered tumour cell phenotype in several other assays performed in vitro (migration in Figure 3, actin dynamics in Figure 4 and CDC42 activity in Figure 5) and in vivo (metastasis and tumour progression, Figure 6). Furthermore, the changes in the spreading phenotype observed in MDA-MB-231 cells in Figure 2 were replicated in PC3 cells (Supplementary Figure S3).

Comment: It is not clear to me why the authors looked at CDC42. Spreading is clearly a Rac phenotype (also CDC42). Why not looking at that GTPase?

Response: We focussed on CDC42 (rather than RhoA or Rac1) based on the paper by Reymond et al 2012. Using MDA-MB-231 and PC3 cells, they showed that tumour cell adhesion to EC required Rac1, RhoA and CDC42; however, depletion of only CDC42 reduced tumour cell TEM.

Using the same cell lines, we found that CD99 inhibits CDC42 activity and accordingly we detected increased tumour cell spreading upon CD99 depletion. In the revised manuscript we show that increased spreading upon CD99 depletion is CDC42 dependent (Figure 5B). Furthermore, as would be predicted from our data and that of Reymond et al, the depletion of CD99 resulted in increased metastasis in vivo (Figure 6).

Interestingly, since our original submission, a paper has appeared in the literature showing that EGFR signalling in MDA-MB-231 cells enhances RhoA and Rac1 activity and this is inhibited by CD99 signalling (Lee et al 2020). We discuss our common findings of an inhibitory role for CD99 in regulating Rho GTPase activity and discuss how CD99 can mediate several key important steps in cancer progression based on its connectivity to Rho GTPases, namely growth factor signalling (RhoA and Rac1: Lee et al) and TEM/metastasis (CDC42; our revised manuscript).

Comment: I would suggest restructuring the paper. Why is the part with angiogenesis included? I would like to have seen some more mechanistic evidence on how CD99 may control CDC42 activity and not one of the other closely related GTPases. And is there a potential GEF involved?

Response: In hindsight we agree that the angiogenesis data is out of place in the manuscript and that it detracts from the main message of the work. We have therefore restructured the manuscript and removed the angiogenesis data.

We have analysed the CD99 depletion phenotype in more detail (in terms of migration, actin dynamics and requirement for CDC42; Figures 3, 4 and 5 respectively). Regarding a candidate GEF, Beta-Pix is implicated in signalling downstream of PKA (also downstream of CD99) and Beta-PIX was recently identified as a GEF regulating the CDC42 dependent metastasis of pancreatic cancer. We discuss Beta-Pix as a candidate GEF, but also point out that there are approximately 40 GEFs documented to regulate CDC42 activity. Identification of the GEF(s) involved requires a more detailed characterisation than we have been able to perform during the pandemic.

Reviewer 2 Comments for the Author:

Comment: 1) The study shows CD99 expression in different breast cancer lines and then uses the metastatic cell line MDA-MB-231 to experimentally demonstrate a role of CD99 in TEM. It is not clear why the authors chose this specific cell line if they were planning to perform gain-of-function assays (i.e. gaining metastatic capabilities) – it would have made more sense to use a low metastastic cell line (e.g. MCF7).

Response: We chose MDA-MB-231 cells for three reasons.

© 2021. Published by The Company of Biologists under the terms of the Creative Commons Attribution License (https://creativecommons.org/licenses/by/4.0/).

i) as now outlined in the results section (with references), this cell line has been used extensively to study the metastasis pathway.
ii) this cell line was used by Reymond et al 2012 to investigate breast cancer TEM in relation to EC adhesion, for the spreading (also called intercalation) assays and to demonstrate a requirement for CDC42 in TEM (in vitro and in vivo).
iii) MDA-MB-231 is representative of triple negative breast cancer (HER2neg, ERneg, PRneg) where lack of treatment options and increased metastasis are a more pressing clinical problem.

In contrast, MCF7 represents ER+ breast cancer and is poorly invasive. Whilst CD99 depletion and increased CDC42 activity might increase this activity (gain of function), invasiveness and metastasis is unlikely to be solely determined by this pathway and MDA-MB-231 with its proven utility in metastasis research was chosen as a more appropriate system, in particular for studying in animal models.

Comment: a) First of all, quantification of the differential expression of CD99 in these systems is required, as the FACS analysis (Fig 1a) does not illustrate well whether there are differences in expression.

Response: We now present both flow cytometry and immunoblotting data comparing CD99 expression in MCF7 and MDA-MB-231. Quantification shows no significant difference in CD99 expression levels between these two cell lines (new Figure 1). We decided to pursue our study in MDA-MB-231 cells as outlined above.

Comment: b) It appears that MCF7 cells express more CD99, which is in line with the proposed role presented in this study. However, this needs to be confirmed appropriately. It will be good to compare expression levels of CD99 between high-metastatic (MDA-231)and low-metastatic cell lines (e.g. MCF7) against normal epithelial cells (e.g. MCF10A).

Response: Expression levels of CD99 are not different statistically significant between MCF7 and MDA-MB-231 (Figure 1) and any differences in invasiveness of these two cell lines does not seem to be simply attributable to CD99 expression. Indeed, we consider it unlikely that differential expression of CD99 expression from cell lines isolated from different breast cancer types (MDA-MB-231; triple negative, MCF7: ER+, PR+) would be sufficient to explain their metastatic behaviour.

Comment: c) If that is the case, it would be appropriate to perform perturbation assays with MCF7 cells to test whether blocking CD99 function leads to the acquisition of TEM and metastatic capabilities.

Response: We have focussed on MDA-MB-231 cells for the reasons discussed above.

Comment: d) The rationale for the inclusion of MDA-BrM in the analysis is not clear. In particular since it appears to have more CD99 expression that the parental cell line, which argues against a critical role in their metastatic potential. In addition, how do the authors reconcile this data with the lower CD99 expression observed in brain mets vs primary tumours (Fig 4f)?

Response: We agree with the referee that inclusion of MDA-BrM was not well justified. The fact that it expresses more CD99 than MDA-MB-231 suggests that differences in CD99 expression do not underlie its preferential metastasis to brain. We have removed this cell line from the revised manuscript, focussing on MDA-MB-231 cells for the reasons discussed above.

Comment: 2) The authors focus in a potential role of Cdc42 downstream of CD99 to explain some of their results. However, modulation of other Rho GTPases (i.e. RhoA, Rac1) may yield similar results. a) It is critical to assess that CD99 does not affect other Rho GTPases.

Response: as noted in response to Reviewer 1, TEM of MDA-MB-231 cells is CDC42 dependent, but Rac1 and RhoA independent (Reymond et al, 2012). We therefore chose to focus on CDC42. As also discussed above, CD99 has subsequently been shown to inhibit RhoA and Rac1 activity downstream of EGFR signalling in MDA- MB-231 (Lee et al, 2021) and in the revised manuscript we discuss how CD99 is coupled to both regulation of proliferation and TEM/metastases via Rho GTPase activity.

© 2021. Published by The Company of Biologists under the terms of the Creative Commons Attribution License (https://creativecommons.org/licenses/by/4.0/).

Comment: b)In addition, the characterisation of the role of CD99 as a potential modulator of cytoskeleton and cell shape is very poor. Changes in F-actin are barely evident and unbiased quantification of F-actin features is required. Is this phenotype only observable in cells seeded on collagen?

Response: We have now performed an unbiased quantification of actin in CD99 depleted cells versus controls. We stained for actin content and measured pixel intensity across siRNA treated cells (new Figure 4 and new supplementary Figure S5). The enhanced spreading phenotype observed upon CD99 depletion occurs when tumour cells are in contact with EC or seeded on gelatin and collagen matrices, but not on fibronectin (new Figure 2A, C). We performed the actin quantification on collagen (new Figure 4B and C) and on fibronectin (new supplementary Figure S5) and found a statistically significant redistribution of actin upon CD99 depletion of tumour cells on collagen but not fibronectin. Importantly, the total amount of actin present was unchanged when CD99 was depleted and the actin redistributed (Figure 4D).

Comment: c)The role of Cdc42 downstream of CD99 needs to be confirmed experimentally. For example, does Cdc42 suppression diminish the effects of loss of CD99 in TEM processes?

Response: We have now performed TEM assays using tumour cells depleted of CD99, CDC42 or both molecules. These results show that the increased spreading phenotype observed upon CD99 depletion is CDC42 dependent (new Figure 5B and C).

Comment: d) It is also very important to highlight that apparently CD99 affects primarily Cdc42 expression/stability, as shown by the increase of total Cdc42 levels. Analyses of Cdc42-GTP/total Cdc42 levels will provide information on whether Cdc42 is more active or there is simply more Cdc42. The authors need to discuss this fact in particular in regards to previous literature, as they suggest that downstream effectors of CD99 (i.e. sAC and PKA, page 17) have been involved in regulating Cdc42 activity – do they affect Cdc42 levels/stability? Does modulating sAC or PKA affect the cytoskeletal features/TEM induced by loss of CD99?

Response: We have now performed a more rigorous analysis of CDC42 activity (using PAK1 pulldowns) and CDC42 absolute levels following CD99 depletion. This includes data generated from the siRNA pool targeting CD99 and the individual siRNAs within this pool. Our results show a significant increase in CDC42 activity upon CD99 depletion with all siRNA molecules (new Figure 5A). There are small increases in total CDC42 levels upon CD99 depletion which are significant when analysed using paired statistical testing (new Figure 5B). In the new manuscript we discuss transcriptional and post-transcriptional mechanisms which regulate CDC42 levels and discuss these in light of our findings and their relationship to the findings of Reymond et al, 2012. Furthermore, we discuss literature demonstrating functional linkage of PKA and CDC42, PKA and the GEF Beta-Pix and the regulation of metastatic activity in pancreatic cancer by a Beta-PIX-CDC42 pathway (Feoktistov et al., 2000; Chahdi et al, 2005; Chernichenko et al, 2020). sAC/PKA activity is known to impact upon actin dynamics (Howe 2004) and interestingly, the PKA pathway has recently been shown to be disrupted in metastatic breast cancer (Paul et al, 2020).

Comment: 3) Overall, the experiments are well designed, but the study requires more rigorous analyses and description. a) Each experiment needs to state the number of biological replicates performed.
b) Quantification of western blots and statistical analysis of band intensities is missing for all the experiments shown. All western blots should be accompanied by quantification of the bands with appropriate replicates and corresponding statistical analyses. Same occurs with FACS analysis.

Response: The number of replicates performed is included in the figure legends and, for some analyses, also in the materials and methods (e.g. for actin quantification). The revised manuscript also includes quantification of blots and flow cytometry data along with statistical analysis.

Comment: c) Generally speaking all the images are not very representative: exemplars in Fig 1B, 5F are not appropriate as they do not show great observable differences –

Response: Figure 1B in the original manuscript has been replaced with a better image in the

© 2021. Published by The Company of Biologists under the terms of the Creative Commons Attribution License (https://creativecommons.org/licenses/by/4.0/).

revised manuscript (new Figure 1C; adhesion of fluorescent, CD99 depleted tumour cells to unlabelled EC). Figure 5F of the original manuscript was angiogenesis assays; this data has been removed from the manuscript as discussed above.

Comment: how do the authors identify individual cells vs cell clumps in the cell adhesion experiments?; Fig S3a is variably seen; bar and bar description are not provided (or barely observable) in most of the images.

Response: Individual cells and cell clumps in the adhesion experiments were distinguished using ImageJ analysis (watershed function) as now described in the materials and methods section (Tumour-EC adhesion assay).

Comment: Finally, metastasis (Fig 4) are hardly seen among the tissue; the authors should consider using outlining the metastatic foci from the rest of the tissue.

Response: We have now used better images of the lung tumours. Staining with Hoechst and detection of human CD99 and Ki67 shows the overlap of CD99 and Ki67 expression in the tumours (new Figure 6D). Furthermore, quantification of Ki67+ cells shows increased tumour cells in the lungs of mice injected with CD99 depleted tumours (new Figure 6E), supporting the in vivo luciferase imaging (new Figure 6A and B and new Supplementary Figure S6).

Comment: d) It is not clear how the authors analyse TEM and intercalation (i.e. Fig 2). Is this all based in cell morphology/area? How do the authors determine if a cell is “spread”? Do the authors correct for cell on/between the endothelial monolayer? Can they identify the cells that have migrated through the monolayer?
For inferring TEM capabilities, it is critical that the authors use more standard assays such as TEM through endothelial monolayers in transwells and count the cells that have successfully transmigrated. Altogether, these analyses (as presented and described) are prone to bias which may affect the conclusions

Response: Image spreading analysis is now described in more detail in the Materials and Methods section (Intercalation and live cell imaging) along with an example analysis mask (Supplementary Figure S7). A threshold based on cell size with a radius of 100 µm was applied. Further filters were applied with a minimal area of 150 µm and a maximum eccentricity of 0.7 (where 0.0 represents a perfect circle). This analysis mask allows discrimination between MDA-MB-231 cells which remain on the apical surface of the EC monolayer and have not undergone TEM, and those which have begun to intercalate and spread, which is indicative of TEM. A limitation of this approach is that migration through the EC monolayer is only inferred and there is no discrimination between those spreading on top of EC or in between the EC cell junctions. However, as the masking does not allow for individual selection of specific cells in the analysis, this strategy allows a unbiased methodology of determining cancer cell spreading on or between endothelial cells. As suggested by the reviewer, we have confirmed changes in TEM activity using the transwell assays (new Figure 2D).

Comment: 4) Key experiments lack proper controls. It is essential that the authors deconvolute the RNAi Smartpool into individual RNAi to discard off-target effects in key experiments.

Response: we have now included data using the individual siRNAs from the Smartpool. This data shows significantly increased migration (Figure 4B and C) and significantly increased CDC42 activity (Figure 5A) when CD99 expression is depleted with the siRNA Smartpool or each of its four constituents. Data showing the levels of knockdown with these siRNAs are shown in Figure 5A and in Supplementary Figure S4.

Comment: 5) When assessing adhesion (Figure 1c and 1f) and spreading (e.g. Figure 5b), the authors need to discuss and provide a reasonable explanation as why in certain time points there are no significant differences between control and experimental points. For example, in Figure 1F there are no significant differences at 120 min but there are at previous time points. This suggests that possibly the efficacy of the process is not altered, just the dynamics.

Response: Figure 1F in the original manuscript is Figure 1E in the revised manuscript. This figure

© 2021. Published by The Company of Biologists under the terms of the Creative Commons Attribution License (https://creativecommons.org/licenses/by/4.0/).

shows that siRNA-mediated depletion of CD99 in tumour cells decreases adhesion to endothelial cells; as the reviewer correctly points out, there are statistically significant differences between si99 and controls at 15, 30 and 60 minutes, but that differences are not significant at 120 minutes. We suggest that this reflects the fact that multiple cell surface receptors participate in adhesion and TEM (e.g. CD99, JAM-A, PECAM/CD31, VE-cadherin, integrins) and that these act at different stages of the process; we cannot address the subtlety of efficacy versus dynamics from this simple adhesion experiment. However, it is clear from this experiment that tumour cell CD99 is required for initial adhesion events, but the requirement is less stringent as the interactions persist (and presumably other adhesion molecules participate in the process). Blocking antibodies (new Figure 1C) confirm the requirement for CD99 in tumour-EC adhesion. CD99 depletion enhances cell spreading (new Figure 2B), with statistically significant events seen at later timepoints. Analysis of the pathway of leucocyte TEM has shown that CD99 interactions are a relatively late event (with PECAM being an early requirement, followed by PVR and then CD99; reference).

Comment: 6) The authors show that adhesion to plastic is not affected by CD99 depletion, but then they assess migration by wound healing in plastic. What about migration in gelatin/collagen/endothelial monolayer where they show that CD99 depletion has an effect?

Response: In the new manuscript, we assess migration in several ways. We have now performed transwell migration assays where the tumour cells must traverse an EC monolayer (new Figure 2D). Furthermore, Xcelligence data (new Fig 3A) provides real time measurements of tumour migration through a modified transwell filter. In addition, we have performed new scratch assays (with the siRNA pool and individual siRNAs from the pool). All of this data assays indicates that CD99 is a negative regulator of migration. This is further supported by the incorporation of actin quantitation on the collagen and fibronectin matrices (new Figure 4 and Supplementary Figure S5).

Comment: 7) For the in vivo assays the authors need to show that CD99 perturbation does not affect survival in circulation and arrest at lung capillaries to discard potential effects independent of TEM. IHC analysis of lungs at early time points (~2h post injection of fluorescently labelled cells) should provide that type of information.

In addition, any potential effect of CD99 in cancer cell growth/survival needs to be discarded more rigorously using proliferation assays or similar. Data showing ki67 staining in vivo is not convincing. In fact, it is not clear how they assess ki67 expression vs ki67-positive cells and its relation with cancer cell growth.

Response: We do not have lung IHC data from an early timepoint. However, in vivo luciferase imaging from a 6h time point indicates that there is not a significant difference in luciferase signal between the control and CD99 depleted cells. However, there is reduced signal in the CD99 depleted tumour cells (p=0.06), as shown in new Figure 6A and Supplementary Figure S6. However, despite the lower efficiency of this initial seeding, the CD99 depleted cells produce bigger tumours over the 4 weeks of the assay. There are three other important pieces of data to consider in interpreting this experiment. First, siRNA treated cells were analysed for cell death (new Supplementary Figure S2A). Second, a crystal violet-based proliferation assay showed no significant difference in proliferation in vitro between control and CD99 depleted cells (new Supplementary Figure S2B) We apologise that this experiment was incorrectly labelled in the original manuscript. Thirdly, we performed transient transfection of CD99 targeting siRNAs which knockdown the CD99 molecule for a few days post transfection; the in vivo model is run over four weeks and tumours detected in the animals using anti-CD99 antibodies (new Figure 6D). As discussed above, we present improved images of the lung tumours; this shows that CD99 and Ki67 overlap (new Figure 6D) hence the proliferating cells in the mouse lung are the human tumour cells. There is a greater tumour burden in the animals injected with the CD99 depleted tumour cells, as shown by quantification of Ki67+ cells (new Figure 6E). We have removed the data on Ki67 intensity as we agree that this is difficult to interpret and adds nothing to the data.

Comment: 8) The analyses of CD99 role in EC are essentially anecdotal without further characterisation. Analyses of cell-cell junction, cytoskeletal features, EC migration etc are required to provide information regarding CD99 function in EC and its relation to TEM.

Response: We have removed the EC experiments from the revised manuscript as stated above.

© 2021. Published by The Company of Biologists under the terms of the Creative Commons Attribution License (https://creativecommons.org/licenses/by/4.0/).

Minor comment: The abstract is not very clear in describing the mechanistic findings, particularly in what concerns CD99 role in cancer cells vs endothelia.

Response: Removal of the EC data from the manuscript and revision of the abstract has, we hope, improved clarity.

Minor comment: -Page 3. This sentence is confusing and needs revision, as it is not clear whether CD99 reduces migration AND tumour growth, or whether reduces migration but increases tumour growth: “[...] in particular in Ewing’s sarcoma where lower CD99 expression results in reduced migration, tumour growth and metastasis”.

Response: this sentence has been revised.

Minor comment: -Since the authors focus on analysis of CD99 in breast and prostate cancer models, the clinical/biological background of this molecule in those settings needs to be introduced in the Background section. In addition, a clear statement describing the use the particular BC and prostate cancer models in this study needs to be reasoned.

Response: we have provided more information in the background section and additional information in the results and discussion.

Minor comment: -As explained before, the authors need to be careful in discussing the results on Cdc42, in particular references to CD99 negatively impacting Cdc42 activity. Data shows that it mainly affects protein expression/stability.

Response: The original manuscript showed effects on CDC42 activity (using CDC42-GTP pulldown assays) and upon total CDC42 levels. In the revised manuscript we have performed the CDC42 activity assays using the deconvoluted siRNA pool and confirm that CDC42 activity is indeed enhanced upon CD99 depletion (new Figure 5A). Levels of total CDC42 do show small increases upon CD99 depletion (new Figure 5A and B) and we discuss this in light of post-transcriptional regulation of CDC42 and transcriptional responses downstream of CDC42 and actin remodelling.

Minor comment: -Figure 1b: what time point is shown?

Response: we apologise for this omission in original Figure 1B. The timepoint for the original image is 30 minutes. We have now replaced this image in the revised version (new Figure 1C) with 30 minutes stated in the legend. The revised manuscript also includes an image of CD99 siRNA depleted tumour cells adhering to EC (new Figure 1E), this image is from the 60 minute time point, also stated in the legend.

<u>Second decision letter</u>

MS ID#: JOCES/2019/240135

MS TITLE: CD99 regulates cancer cell transendothelial migration via CDC42 and actin remodelling

AUTHORS: Aarren J Mannion, Adam F Odell, Alison Taylor, Pamela F Jones, and Graham P Cook
ARTICLE TYPE: Research Article
We have now reached a decision on the above manuscript.

To see the reviewers' reports and a copy of this decision letter, please go to: https://submit-jcs.biologists.org and click on the 'Manuscripts with Decisions' queue in the Author Area. (Corresponding author only has access to reviews.)

Please address the comment from Reviewer 1. I would be pleased to see a revised manuscript

© 2021. Published by The Company of Biologists under the terms of the Creative Commons Attribution License (https://creativecommons.org/licenses/by/4.0/).

We are aware that you may be experiencing disruption to the normal running of your lab that makes experimental revisions challenging. If it would be helpful, we encourage you to contact us to discuss your revision in greater detail. Please send us a point-by-point response indicating where you are able to address concerns raised (either experimentally or by changes to the text) and where you will not be able to do so within the normal timeframe of a revision. We will then provide further guidance. Please also note that we are happy to extend revision timeframes as necessary.

Please ensure that you clearly highlight all changes made in the revised manuscript. Please avoid using 'Tracked changes' in Word files as these are lost in PDF conversion.

I should be grateful if you would also provide a point-by-point response detailing how you have dealt with the points raised by the reviewers in the 'Response to Reviewers' box. Please attend to all of the reviewers' comments. If you do not agree with any of their criticisms or suggestions please explain clearly why this is so.

Reviewer 1

Advance summary and potential significance to field

The revision is a much better version compared to the first submission. it shows that CD99 on tumor cells (specifically MDA-MB-231 cells) can regulate the vascular passage of these cells across the endotheluim.

Comments for the author

I am satisfied with the changes the authors have made. I think they addressed my concerns. Only one thing is that I would like to see that they stress better that it is CD99 on the tumor cell that they investigated, not the CD99 on the EC. This should be mentioned in the abstract, perhaps even in the title. and can be repeated in the discussion, can just be one sentence extra.

Reviewer 2

Advance summary and potential significance to field

The study “CD99 regulates cancer cell transendothelial migration..." by Mannion et al investigates molecular mechanisms that regulate a critical step in cancer metastasis: transendothelial migration required for cancer cell extravasation and colonization of secondary sites. Understanding the mechanisms that control the ability of cancer cells to invade and metastasise is critical for the development of new therapeutic strategies and monitoring/diagnostic tools that may eventually benefit patients. In addition, it will inform of the fundamental biology underpinning cell migration that may be useful in other disciplines (immunology, development etc).
The work presented here is significant because it identifies a new regulator of cancer metastasis and provides clues about the molecular mechanisms downstream of CD99.

Comments for the author

The authors have addressed all my previous comments and I would like to sincerely congratulate them for their efforts and work. I acknowledge it must have been tough given the circumstances.

Second revision

Author response to reviewers' comments

Thank you for the very positive reviews of our revised manuscript.

© 2021. Published by The Company of Biologists under the terms of the Creative Commons Attribution License (https://creativecommons.org/licenses/by/4.0/).

Reviewer 2 is satisfied with the revision.

Response: we are grateful and appreciative of the reviewers' comments.

Reviewer 1 is also satisfied with the changes but recommends that we make it clear that our results refer to cancer cell CD99 (and not CD99 on the endotheium).

Response: We have changed the title, abstract and text to reflect this (highlighted in yellow in the manuscript).

I hope that these changes now make the manuscript acceptable for publication.

Third decision letter

MS ID#: JOCES/2019/240135

MS TITLE: Tumour cell CD99 regulates transendothelial migration via CDC42 and actin remodelling

AUTHORS: Aarren J Mannion, Adam F Odell, Alison Taylor, Pamela F Jones, and Graham P Cook
ARTICLE TYPE: Research Article

I am happy to tell you that your manuscript has been accepted for publication in Journal of Cell Science, pending standard ethics checks.

© 2021. Published by The Company of Biologists under the terms of the Creative Commons Attribution License (https://creativecommons.org/licenses/by/4.0/).
